# SMC ensures efficient chromosome replication and *oriC* positioning during *Streptomyces* spore germination

**DOI:** 10.1038/s41598-026-43107-5

**Published:** 2026-03-15

**Authors:** Katarzyna Pawlikiewicz, Agnieszka Strzałka, Agnieszka Nurek, Magdalena Donczew, Anna Gierlikowska, Martyna Gongerowska-Jac, Marcin J. Szafran, Dagmara Jakimowicz

**Affiliations:** 1 Department of Molecular Microbiology, Faculty of Biotechnology, University of Wroclaw, Wroclaw, Poland; 2 Present Address: Max Planck Institute for Terrestrial Microbiology, Marburg, Germany; 3 Present Address: Oklahoma Medical Research Foundation, Oklahoma City, USA

**Keywords:** Condensins, Chromosome replication, Bacterial chromosome organization, Spore germination, Genetics, Microbiology, Molecular biology

## Abstract

Bacterial chromosomes are organized by condensins, such as Structural Maintenance of Chromosomes (SMC) proteins. In *Streptomyces*, a genus of sporulating bacteria, SMC proteins align chromosomal arms and promote efficient compaction of chromosomal DNA during spore formation. We hypothesized that disrupting nucleoid architecture by deleting the *smc* gene would affect the positioning of the origin of replication (*oriC*) or the process of chromosome replication during spore germination. To test this hypothesis, we conducted marker frequency analyses and microscopy studies to observe the positioning of labelled *oriC* and replisomes in both wild type and Δ*smc* backgrounds. Additionally, we investigated the positioning of three chromosomal loci in early vegetative cells. Our results indicate that the deletion of *smc* impairs chromosome replication and hinders germ tube development. Furthermore, detailed analysis of chromosome organization revealed that, in the absence of SMC, the *oriC* region becomes mispositioned within the nucleoid. These findings underscore the important role of SMC in maintaining nucleoid architecture during the early growth stages of *Streptomyces*.

**Supplementary Information:**

The online version contains supplementary material available at 10.1038/s41598-026-43107-5.

## Introduction

The DNA molecule that makes up the bacterial chromosome is highly organized. The chromosomal DNA is supercoiled and arranged into loops^1–3^. This organization allows for the compaction of genetic material within the limited space of the cell and facilitates efficient DNA transaction, including replication, segregation, and transcription^1,4–6^.

Specific regions of the chromosome, particularly the origin (*oriC*) and terminus (*ter*) of replication, are precisely positioned within bacterial cells, and their placement significantly influences the overall arrangement of the chromosome^1,7–9^. The location of the *oriC* region varies depending on the bacterial species and their growth modes. In many bacteria, such as *Caulobacter crescentus*, *Mycobacterium smegmatis*, *Myxococcus xanthus*, and *Vibrio cholerae*, the *oriC* region is anchored at or near one of the cell poles^10–15^. This positioning results in the location of the terminus region near the opposite pole and close alignment of the chromosomal arms. Such longitudinal organization of the chromosome is also referred to as *ori-ter*. In contrast, slow-growing *Escherichia coli* has its *oriC* positioned at the centre of the cell, with the left and right chromosomal arms occupying opposite halves (an *arm-ori-arm* arrangement). However, in fast-growing *E. coli*, the *oriC* region is situated near one of the cell poles^1,4,10,16^. Similar changes in chromosome organization and *oriC* positioning that depend on the cell cycle stage have also been observed in *Bacillus subtilis*^17^. Studies utilizing chromosome conformation capture experiments have emphasized the close alignment of chromosomal arms in an *ori-ter* arrangement^2,18^.

Chromosome organization is orchestrated by a set of proteins, among which condensins play a pivotal role^19^. Condensins are a family of proteins found across all domains of life^20^. Most bacterial species possess the SMC protein (Structural Maintenance of Chromosome), except for *E. coli* and other Gamma- and Deltaproteobacteria, which encode a functional homologue of SMC, MukB^21,22^. Both SMC and MukB exhibit the conserved elongated structure characteristic of condensins and share their ability to dimerise. Their rod-shaped architecture is defined by a coiled-coil domain that separates the globular head domain from the hinge region that forms the dimerisation domain^23,24^. Condensins facilitate DNA compaction through a mechanism called loop extrusion, which depends on conformational changes within the dimer^20,25–27^. Analyses of chromosome conformation suggest that SMC proteins are crucial for establishing long-distance contacts within the chromosome, which is consistent with their function as loop extruders^28,29^. Cytological studies have also shown that the elimination of condensins results in the decompaction of chromosomal DNA and may lead to the formation of anucleate cells, the fraction of which depends on the bacterial species and growth conditions^21,30^. Interestingly, studies on *E. coli* or *B. subtilis* did not indicate that the absence of SMC affects chromosome replication. However, recently, the lack of SMC was reported to impact replisome progression in *C. crescentus*^31^. While the role of SMC in the compaction of chromosomal DNA is well established, its impact on DNA-dependent processes, such as replication and transcription, remains to be fully elucidated.

SMC is recruited near the *oriC* region by the partitioning protein ParB^32,33^. ParB is responsible for the local organization of the *oriC* proximal region and, in cooperation with its partner protein ATPase ParA, for its accurate positioning in the cell^34–37^. ParB interacts with numerous DNA sequences, known as *parS* sites, and through nonspecific interactions with *parS*-proximal DNA, forms large nucleoprotein complexes called segrosomes^38–41^. Soon after the initiation of chromosome replication, newly duplicated segrosomes are separated due to their interaction with ParA. SMC cooperates with ParB in *oriC* segregation^17,42^. Also, in *E. coli* (which lacks ParB homologues), the elimination of MukB disrupted *oriC* segregation^43,44^. The actual mechanism by which condensin contributes to *oriC* location is still under debate, and the question of how condensins affect *oriC* positioning within the nucleoid remains unresolved.

*Streptomyces* are mycelial, sporulating soil bacteria. They utilise SMC for chromosome remodelling during differentiation and effective DNA compaction in spores^45–47^. The complex life cycle of *Streptomyces* starts with spore germination. The germ tube emerges from the spore and extends apically to form a vegetative hyphal cell^48,49^. The elongated cells branch but remain unseparated upon cell division, developing a hyphal network. During sporulation, sporogenic cells undergo multiple divisions to form chains of exospores. Upon maturation, spores are dispersed and germinate at optimal growth conditions^50,51^.

Spores are the only unigenomic cells in *Streptomyces*. During germination, chromosomal replication initiates in the spore before the germ tube emerges^52,53^. This initial replication process delivers multiple copies of the chromosome, which are directed to the germ tube. Multiple chromosomal copies replicate asynchronously within elongated hyphal cells^52,54,55^. To facilitate the effective apical elongation of hyphal cells and their branching, the apical chromosome remains anchored at the tip of the hypha^56^. This anchorage is provided by the ParB protein, which, like in other bacteria, forms a complex near the *oriC* region. The ParB complex interacts with the ParA protein that is associated with the hyphal tip^56,57^. In sporogenic cells, ParA and ParB help segregate the multiple chromosomal copies to create unigenomic prespore compartments. At this stage, chromosome segregation is tightly coordinated with multiple cell divisions and is accompanied by significant compaction of the chromosomal DNA^45,58^. Chromosome conformation analyses have shown that the structure of the chromosomes changes during the life cycle^46,59–61^. In the later stages of vegetative growth, chromosomes primarily exist in an open conformation with limited interactions between their arms. During sporulation, these interactions gradually increase, leading to closer proximity of the chromosomal arms in the spores. Our earlier studies showed that in *S. venezuelae*, the SMC protein plays a crucial role in aligning the chromosomal arms during sporulation and in compacting the DNA within the spores. When SMC is eliminated, enlarged spores are formed that contain decompacted nucleoids^46^. However, the effect of SMC on the positioning of the *oriC* region in spores and during germination remains unknown.

Having established that SMC contributes to efficient DNA organization in spores, we investigated how the absence of SMC impacts DNA-dependent processes and overall nucleoid architecture during *S. venezuelae* spore germination. To address this question, we analysed whether SMC affected chromosome replication during germination and vegetative cell elongation. Next, using strains with labelled *oriC*, we microscopically examined positioning of this chromosomal region during spore germination in the presence and absence of SMC. Additionally, we determined how SMC-dependent chromosome organization contributes to *oriC* positioning within the nucleoid and its anchoring at the pole of the elongating vegetative cell.

## Results

### The loss of SMC affects chromosome replication, impacting spore germination in* S. venezuelae*

SMC plays a crucial role in chromosome compaction during the sporulation of *Streptomyces venezuelae*^46^. However, condensin levels remain constant throughout the entire life cycle, including during spore germination^62^. This raises the question of whether removal of SMC—and the resulting disruption of SMC-dependent chromosome organization—impacts spore germination. To explore the influence of SMC-dependent chromosome organization on spore germination, we analysed this process in three strains: the wild type *S. venezuelae* strain (WT), a strain with *smc* deletion (TM010), and a complemented *smc* deletion strain. Earlier analyses of this complemented strain (Δ*smc*, *smc-flag* (TM019) with the *smc-flag* gene in the integrating plasmid) focused on nucleoid compaction in spores and suggested that the SMC-FLAG protein was functional^46^.

**Fig. 1:**
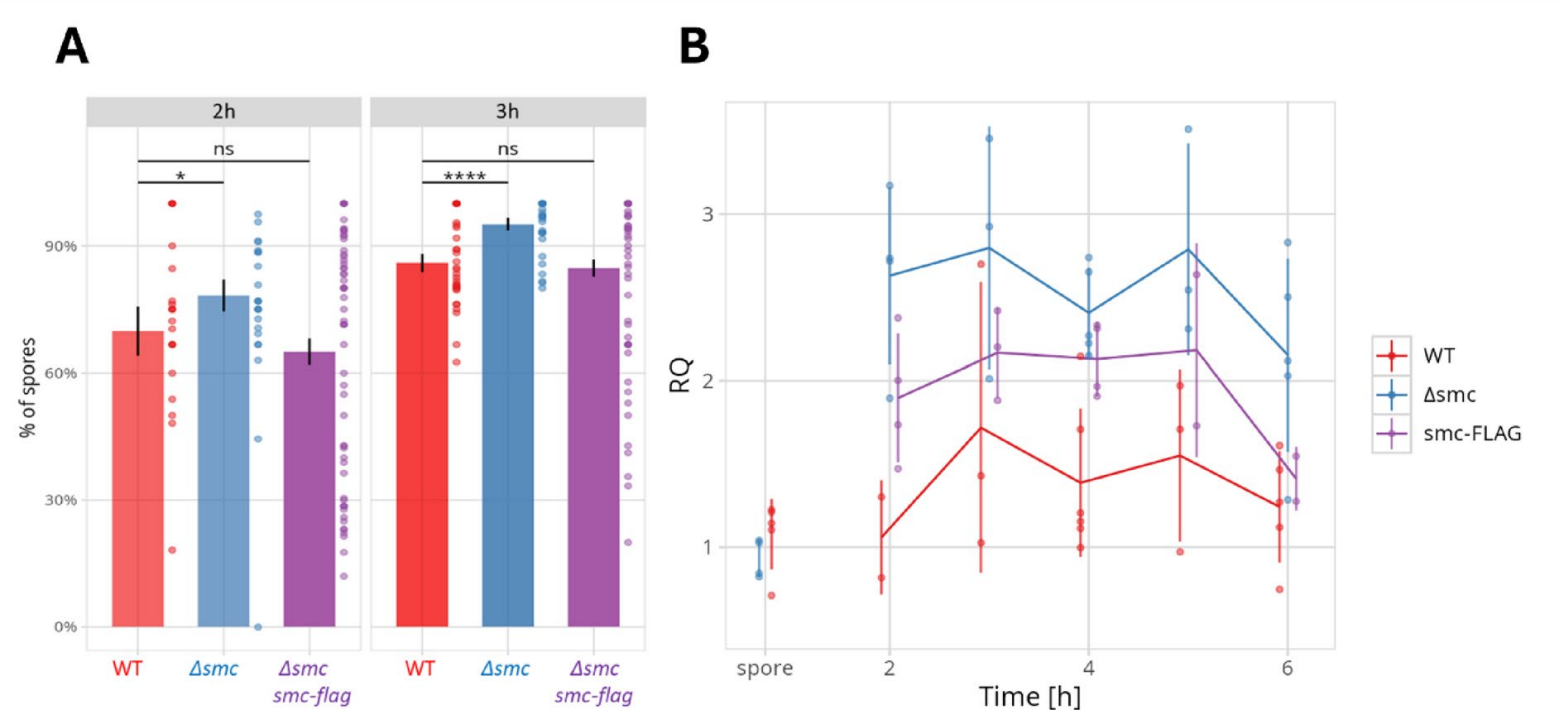
The SMC elimination affects the *S. venezuelae* spore germination and the chromosome replication rate. The rate of spore germination and replication was analysed in wild type (WT), *smc* deletion (*smc*, TM010), and complemented *smc* deletion strain (*smc*, *smc-flag*, TM019) (**A**) The fraction of spores that developed the germ tube after 2 and 3 h of cultivation in liquid medium (MYM) based on microscopy analyses. Statistical analysis using equality of proportions hypothesis test was carried out based on microscopy images collected in 2 independent experiments for 252 (2 h) and 1003 (3 h) spores of WT strain, 483 (2 h) and 880 (3 h) spores of TM010 strain and 954 (2 h) and 1292 (3 h) spores of TM019 strain. Each data point represent the value obtained from single microscopy image. (**B**) Marker frequency analyses – determination of *ori/arm* ratio by qPCR on the chromosomal DNA isolated from specific time points of cultivation (2–6 h and 28 th hour - spores) of analysed strains. Vertical lines indicate the standard deviation. The data were obtained in 2–6 biological repeats.

To evaluate the germination rate, we used microscopy to estimate the fraction of spores that developed a germ tube after 2 and 3 h of incubation in liquid medium. In the absence of SMC, the fractions of germinating spores were slightly elevated —78% and 95% at 2 and 3 h in the Δ*smc* strain, compared to 70% and 86% in the wild type strain, respectively (Fig. 1A). The complemented strain displayed germination rates similar to those of the wild type, with 65% and 85% of spores germinating at 2 and 3 h, respectively (Fig. 1A). These findings indicate that the lack of SMC slightly induces germination, while the reintroduction of the *smc-flag* gene reduces this effect.

Previous studies have indicated that chromosome replication begins in spores prior to the formation of the germ tube^52,53^. Notably, at the stage when the germ tube emerges, the germinating spores contain multiple copies of chromosomes^56^. To investigate whether the observed slight induction of germination in the absence of SMC was related to its impact on chromosome replication, we conducted marker frequency analyses during the germination process of the wild type, *smc*, and complemented strains. To this end, the *ori/arm* ratio was determined using the chromosomal DNA isolated from the culture incubated for 2–6 h as compared to chromosomal DNA isolated from spores (28 h culture)^62^. In spores, the *ori*/*arm* ratio was equal 1 in wild type and *smc* strain. In the second hour of spore incubation, the *ori/arm* ratio averaged 1.1 in the wild type strain, while in the strain lacking SMC, this ratio significantly increased to approximately 2.6. In the 3rd hour of incubation, in the wild type strain, the *ori/arm* ratio rose to about 1.5 likely reflecting the increasing number of replicating chromosomal copies and remained at this level in the course of analysis (up to the 6th hour). In the absence of SMC, this ratio remained consistently close to 2.5 throughout the analysed period, indicating altered replication rate. In the complemented strain, the *ori/arm* ratio was approximately 1.9, which fell between the values observed in the wild type and *smc* strains, indicating that SMC-FLAG is not fully functional in restoring the replication rate (even though it was efficient in chromosome compaction, as indicated earlier^46^. The increased *ori/arm* ratio seen in the absence of SMC may be partially attributed to a somewhat higher proportion of germinating spores in the *smc* deletion strain compared to the wild type. However, the two-fold increase in the *ori/arm* ratio in the 2nd hour of cultivation exceeds what would be expected based solely on the elevated germination rates (10% increase), suggesting that there may be an over-initiation of replication or disturbances in the replication process, potentially resulting from chromosome disorganization.

Finally, we considered that SMC dependent chromosome organization in spores could also affect transcription. To check such possibility, we analysed the activity of selected promoters of genes encoding transcriptional regulators. These genes, namely *crp* (encoding the global transcriptional regulator), *prs* (encoding the antisigma factor), *sigB* (encoding the alternative sigma factor), *bldD* (encoding the global transcriptional regulator) and *bldG* (encoding the anti- antisigma factor), were selected based on published analyses that indicated their activity during germination^63–66^. Promoters of these genes were cloned into plasmid carrying the *luxCDEAB* reporter genes and the obtained reporter plasmids were integrated into the chromosomal attachment site in wild type and *smc* strains. The activity of *lux* reporter genes under the control of the selected promoters was monitored during germination in liquid cultures. These analyses showed that the absence of SMC altered the transcriptional activity of some of analysed promoters (*crp*,* prs* and *sigB*) in a promoter-dependent manner **(Fig. ****S1****)**. Taking into account that promoter activity was analysed in the same chromosomal context, these analyses indicate a potential impact of SMC-dependent chromosome organization on transcriptional regulatory networks.

In summary, we determined that eliminating SMC induces spore germination, while also affecting chromosome replication and possibly modifying transcriptional activity during this process.

### The absence of SMC delayed the migration of chromosome from spore to the germ tube

**Fig. 2:**
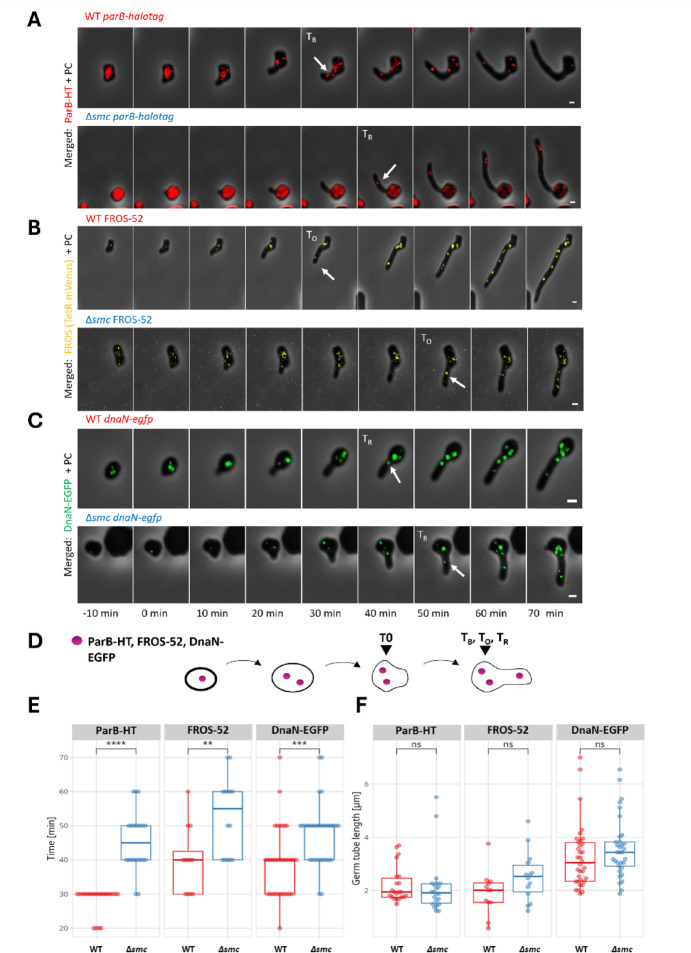
Development of the germ tube and its population with the chromosome is delayed by* smc* deletion. (**A**) Representative images taken during time-lapse analyses of spore germination showing visualisation of ParB-HT stained with Janelia Fluor-549 (red) overlaid on phase contrast (PC) images (grey) in the wild type (KP006, upper panel) and *smc* background (KP007, lower panel). Separate channels are shown in Fig. S3. (**B**) Representative images taken during time-lapse analyses of spore germination showing visualisation of *oriC* - FROS-52 marked by TetR-mVenus (yellow) overlaid on phase contrast (PC) images (grey) in the wild type (KP012, upper panel) and *smc* background (KP015, lower panel). Separate channels are shown in Fig. S4. (**C**) Representative images taken during time-lapse analyses of spore germination showing visualisation of replisome marked by DnaN-EGFP (green) overlaid on phase contrast (PC) images (grey) in the wild type (MD070, upper panel) and *smc* background (KPAG01, lower panel). Separate channels are shown in Fig. S6. (**A**,** B**), and (**C**); 0 min - time of the germ tube emergence, time of ParB-HT focus appearance (T_B_), FROS-52 focus appearance (T_O_), and replisome appearance (T_R_) in the germ tube are marked with the arrow. Scale bar: 1 μm. (**D**) Schematic representation of the analysed time between time of germ tube emergence (T0) and the time of ParB-HT focus (T_B_), FROS-52 focus (T_O_) and replisome (T_R_) appearance in the germ tube (**E**) Boxplot analyses of the time of ParB-HT focus (T_B_), FROS-52 focus (T_O_) and replisome (T_R_) appearance in the germ tube. (**F**) Boxplot analyses of the germ tube length at the time of ParB-HT focus (T_B_), FROS-52 focus (T_O_), and replisome (T_R_) appearance. Boxplots in E and F show median with first and third quartile, while the lower and upper “whiskers” extend to the value no further than 1.5 * IQR (interquartile range) from the “hinge”. Statistical analyses were performed based on data collected for 22 hyphae from KP006 and KP007 strains, for 20 hyphae from each KP012 and KP015 strain, and for 30 hyphae from the MD070 strain and the KPAG01 strain for two biological replicates of the experiment for each strain. Statistical analyses were carried out using the two-sided Wilcoxon test (p-value ≤ 0.01 (**), p-value ≤ 0.001 (***), ≤ 0.0001 (****), ns – no statistical significance).

Next, we set out to test if the disturbed chromosome organization in spores resulting from the absence of SMC^46^ and altered patterns of chromosome replication described above could affect chromosome targeting to the germ tube. To this end, we marked the *oriC*-proximal region in the *S. venezuelae* wild type and *smc* backgrounds using two approaches. The strains producing ParB-HaloTag (KP006 - control strain *parAB parAB-HT* and KP007 - *smc* *parAB parAB-HT*^62^ were used to visualise ParB complex formed at multiple *parS* sites spanning 200 kbp from *oriC* region (Fig. 2A). Additionally, to exclude possibility that visibility of ParB complex during germination was affected by the deletion of *smc*^62^, we used the fluorescent reporter operator system (FROS) to mark regions in three different distances 52, 112 and 353 kbp from *oriC* (FROS-52, FROS-113 and FROS-353, respectively) in the wild type and *smc* strains **(**Fig. 2B**)**. The integrative pOJ_FROS plasmids used for FROS labelling contained 49 *tetO* repeats and the *tetR-mvenus* gene under the constitutive p14 promoter. In all FROS strains, TetR-mVenus formed bright foci in vegetative hyphae only in the presence of *tetO* operators. In the absence of *tetO* or in the presence of anhydrotetracycline, which diminishes TetR binding to its operator sites, the FROS foci were either significantly smaller or fully diffused (Fig. S2A, B). The number and distribution of FROS-52 foci were similar to those of ParB-HT complexes (Fig. S2C), but the presence of TetR-mVenus-*tetO* complex somewhat affected the growth of FROS strains (Fig. S2D). The levels of TerR-mVenus were not affected by the deletion of *smc* or the location of the pOJ_FROS insertion (Fig. S2E). The spore germination of FROS-52 and ParB-HT strains was analysed by time-lapse microscopy. We observed spore swelling followed by the emergence and extension of the germ tube. The fluorescence focus appeared in the germ tube after a certain period of time. We measured the time between the clear emergence of the germ tube (set as time 0) and the appearance of the *oriC* signal in the germ tube.

In the wild type control strain (KP006), ParB-HT complex was detected in the germ tube approximately 28 ± 1.8 min (± 95% confidence interval) after the germ tube emerged, which was regarded as reference time point (Fig. 2A, E, Fig. S3A). Deletion of *smc* (KP007) significantly delayed the appearance of ParB-HT focus in the germ tube as compared to wild type control – the complex was visible 45 ± 3.8 min after emergence of the germ tube (p-value = 2.728e-08, two-sided Wilcoxon test) (Fig. 2A, E, Fig. S3B). The FROS-52 focus in the wild type control strain (KP012) was visible in the germ tube approximately 40 ± 6.1 min after its emergence (Fig. 2B, E, Fig. S4A). The absence of SMC (KP015) visibly delayed the appearance of the FROS-52 signal in the germ tube as the complex was detected in 53 ± 6.6 min after germ tube development (p-value = 0.008, Wilcoxon test two-sided) (Fig. 2B, E, Fig. S4B). The time difference in complex appearance between ParB-HT strains and FROS strains is likely due to differences in their growth rates. Markedly, the length of the germ tube at the time of *oriC* signal appearance (ParB-HT or FROS-52) was not significantly altered by *smc* deletion (for ParB-HT, p-value = 0.31; for FROS-52, p-value = 0.09, Wilcoxon test two-sided) (Fig. 2A, B, F). This observation suggests that the delayed detection of chromosomal *oriC* in the germ tube, in the absence of SMC, may be associated with slower germ tube extension.

Next, we utilized the fluorescently-marked replisome as an additional marker to confirm the presence of the chromosome in the germ tube and analysed whether the absence of SMC affected the timing of replisome appearance in the germ tube. To this end, we used strains producing a DnaN-EGFP fusion (*dnaN-egfp* in its native chromosomal *locus*), allowing visualisation of the replisomes in germinating spores of the wild type control and *smc* strains (MD070 and KPAG01). The production of DnaN-EGFP was confirmed by Western blotting, which also showed that *smc* deletion did not affect DnaN-EGFP levels (Fig. S5A). Notably, the growth rate of mutant strains producing DnaN-EGFP instead of the wild type protein was affected by the fusion; however, these analyses confirmed the functionality of DnaN-EGFP (Fig. S5B). Spore germination of strains producing DnaN–EGFP was analysed by time-lapse microscopy to determine the time between germ tube emergence and appearance of the replisome, as described above.

The time-lapse analyses revealed that in the control strain (MD070), the first replisome in the germ tube appeared 39 ± 3.5 min after germ tube emergence (Fig. 2C, E, Fig. S6A). This time was similar to the time of FROS-52 focus appearance in the germ tube (40 ± 6.1 min), reinforcing our analyses. Deletion of *smc* delayed the appearance of the replisome in the germ tube in comparison to the wild type control (48 ± 3.2 min, p-value = 0.0004, two-sided Wilcoxon test) (Fig. 2C, E, Fig. S6B). Measuring the length of the germ tube at the time of replisome appearance showed that it was similar in the wild type control and Δ*smc* (p-value = 0.18 two-sided Wilcoxon test) (Fig. 2F**)**. These analyses indicated that the absence of SMC delayed the initiation of chromosome replication in the germ tube supporting earlier observed delayed migration of the chromosome from spore into germ tube.

Taken together, time-lapse microscopy analyses of chromosomal *oriC* during germination show that the lack of SMC delays the migration of chromosomes into the germ tube and its extension. This observation could be regarded as contradictory to the above-described over-initiation of chromosome replication. However, the delayed appearance of chromosomal *oriC* in the hyphal cell could result from inefficient DNA replication. Alternatively, aberrant nucleoid architecture in the absence of SMC may disturb *oriC* positioning and hinder efficient targeting of *oriC* to the germ tube.

### The absence of SMC impedes the chromosome multiplication in the early vegetative cell

Having established that the absence of SMC affects chromosome replication during germination and slows development of a germ tube, we set out to analyse if the lack of SMC also had an impact on the number of the chromosomes in the young vegetative hyphae. To this end, based on the time-lapse microscopy experiments, we measured the length of the extending hypha for the first 3 h of growth. Next, we counted the number of *oriC*s (marked by ParB-HT or FROS-52) and the number of replisomes (DnaN-EGFP complexes) relative to hyphal length in the wild type control and *smc* strains.

**Fig. 3:**
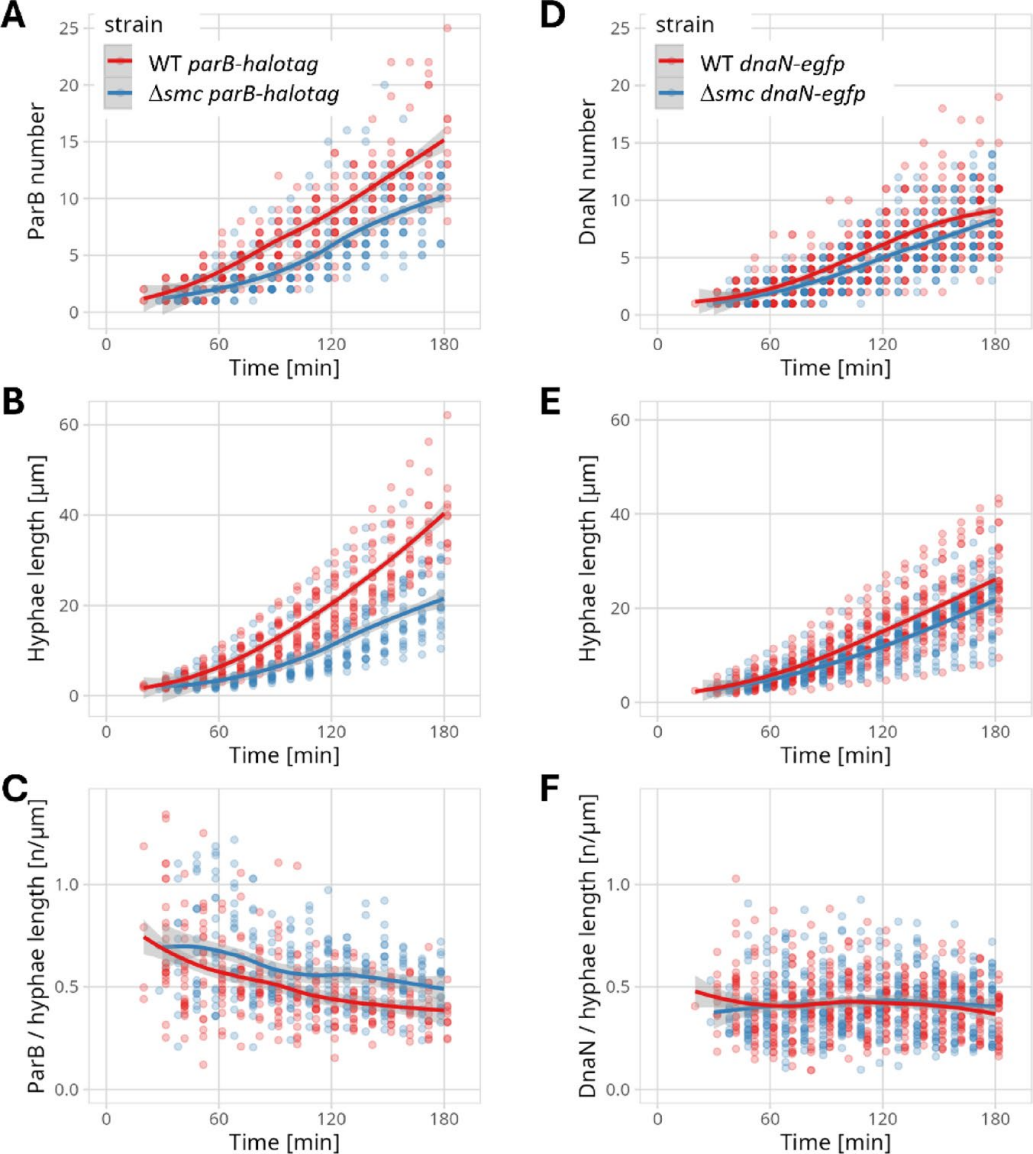
The absence of SMC affects the number of ParB-HT and DnaN-EGFP complexes in the vegetative cell and lowers the cell extension. (**A**). The number of ParB-HT complexes in the vegetative cell, (**B**). the length of the hyphal cell, and (**C**). the density of the ParB-HT complexes (number of ParB-HT complexes per 1 μm of cell length) in the wild type (KP006) and *smc* (KP007) background during the time-lapse experiment analysing hyphal growth since germination (**D.**) The number of DnaN-EGFP complexes in the vegetative cell, (**E**). the length of the hyphal cell, and (**F**). the density of the DnaN-EGFP complexes (number of DnaN-EGFP complexes per 1 μm of cell length) in the wild type (MD070) and *smc* (KPAG01) background during the time-lapse experiment. Statistical analyses were carried out based on data collected for 22 hyphae of KP006 and KP007 strains, for 20 hyphae of KP012 and KP015 strains, and for 30 hyphae of MD070 and KPAG01 strains. The data came from two biological replicates of the experiment for each strain.

The number of ParB-HT complexes increased steadily while the vegetative cell extended, but in the absence of SMC, both the number of ParB-HT complexes and the length of the cell increased more slowly than in the control strain (Fig. 3A, B**)**. Based on the number of ParB-HT complexes and the length of the hyphal cell, we calculated density of *oriC*s. This density was slightly higher in absence of SMC than in the control strain - in the 1st hour of growth, 0.74 +/- 0.12 ParB-HT complex per µm in *smc* strain and 0.56 +/- 0.07 in the wild type, while in 2nd hour of growth, 0.57 +/- 0.07 complex per µm and 0.43 +/- 0.06, in *smc* and wild type strain respectively (Fig. 3C). The higher density of ParB-HT in the absence of SMC corroborates the increased replication rate determined by marker frequency analysis (Fig. 1C). Markedly, the analysis of FROS strains did not indicate a difference in the growth rate and number of FROS-52 complexes in between the wild type and *smc* background (Fig. S7). We infer, that this discrepancy may result from impaired FROS strains growth.

Next, we performed similar time-lapse microscopy-based analyses for strains producing DnaN-EGFP in the wild type and *smc* backgrounds. These strains also showed a lower number of replisomes in the absence of SMC than in the wild type background, accompanied by a slower hyphal elongation; however, the difference in growth rate was less pronounced (Fig. 3D, E). The overall density of DnaN-EGFP complexes was the same (approximately 0.4 complex per µm in both analysed strains) (Fig. 3F), indicating that the number of ongoing replications may be similar in both strains.

Combined, these analyses showed that the absence of SMC slowed the multiplication of chromosomal copies, which may be reflected in slowed hyphal extension. Notably, the increased density of ParB-HT complexes, but not of replisomes, in the absence of SMC indicates an increased number of *oriC*s and is consistent with an increased *ori/arm* ratio, reinforcing potential over-initiation or slowed replication progress.

### The absence of SMC disrupts the* oriC* region positioning relative to the nucleoid and cell pole

**Fig. 4:**
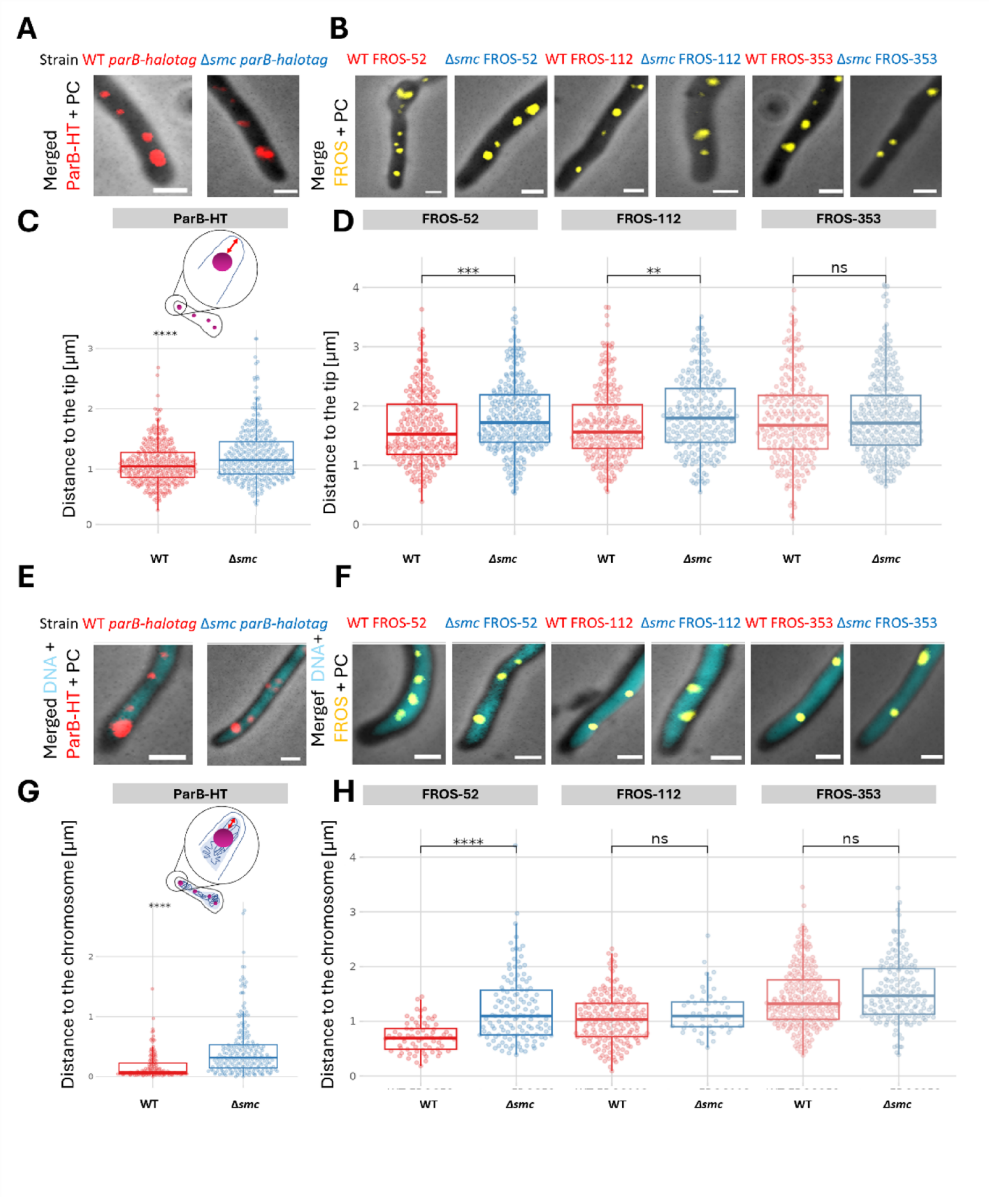
The absence of SMC alters the positioning of* oriC* in relation to the nucleoid and the tip of the* S. venezuelae* young vegetative cell. (**A**). Representative microscopic images showing the apical ParB-HT complex in the wild type (KP006) and *smc* (KP007) background. The images show the fluorescence of ParB-HT stained with TMR *direct ligand* (red) overlaid on a brightfield hyphae image (phase contrast - grey). (**B**). Representative microscopic images showing the apical FROS complex in the wild type: KP012 (WT, FROS-52), KP013 (WT, FROS-112), KP014 (WT, FROS-353), and *smc*: KP015 (Δ*smc*, FROS-52), KP016 (Δ*smc*, FROS-112), KP017 (Δ*smc*, FROS-353) background. The fluorescence of TetR-mVenus (FROS - yellow) is overlaid on a brightfield image (phase contrast - grey). (**C**). Analyses of the distance between the apical ParB-HT complex and the hyphal tip in the wild type (KP006) and Δ*smc* (KP007) background. (**D.**) Analyses of the distance between the apical FROS-52, FROS-112, and FROS-353 complexes and the hyphal tip in the wild type (KP012, KP013, KP014, respectively) and *smc* (KP015, KP016, KP017, respectively) backgrounds. Statistical analyses were conducted based on data collected for 330 ParB-HT complexes in the KP006 and KP007 strains and for 230 TetR-mVenus complexes for the KP012, KP013, KP014, KP015, KP016, and KP017 strains. (**E**) Representative microscopic images showing the apical ParB-HT complex in the wild type (KP006) and Δ*smc* (KP007) background. The images show the fluorescence of ParB-HT stained with TMR *direct ligand* (red) overlaid on Hoechst33342-stained DNA (blue) and brightfield hyphae image (phase contrast - grey) (**F**). representative microscopic images showing the apical FROS complex in the wild type: KP012 (WT, FROS-52), KP013 (WT, FROS-112), KP014 (WT, FROS-353), and *smc*: KP015 (Δ*smc*, FROS-52), KP016 (Δ*smc*, FROS-112), KP017 (Δ*smc*, FROS-353) background. The fluorescence of TetR-mVenus (FROS - yellow) is overlaid on Hoechst33342-stained DNA (blue) and brightfield hyphae image (phase contrast - grey). (**G**). Analyses of the distance of the apical ParB-HT complex to the apical edge of the nucleoid in vegetative hyphae of the control strain (KP006) and *smc* (KP007). (**H**). Analyses of the distance between the apical FROS-52, FROS-112, and FROS-353 complex, and the apical edge of the nucleoid in the vegetative hyphae of the wild type control (KP012, KP013, KP014, respectively) and *smc* strains (KP015, KP016, KP017, respectively). A-G- Hyphae from 5-hour liquid cultures were analysed. Statistical analyses were conducted based on data collected for 150 ParB-HT complexes in KP006 and KP007 strains and 150 TetR-mVenus complexes in KP012, KP013, KP014, KP015, KP016, and KP017 strains in two independent biological replicates. In the statistical analysis, the two-tailed Student’s t-test (**C**) and two-sided Wilcoxon test (**D**,** G**,** H**) were used (p-value ≤ 0.01 (**), ≤ 0.001 (***), ≤ 0.0001 (****), ns – no statistical significance). A, B, E, and F scale bar: 1 μm. Separate channels are shown in Fig. S8.

In *Streptomyces* the *oriC* region of the apical chromosome is anchored at the tip of the hyphal cell^56^. Here, we aimed to determine whether the absence of the SMC protein affects the positioning of the apical *oriC*. To investigate this, we used fluorescence microscopy images of the young vegetative cells (5 h of cultivation in liquid medium) to measure the distance between the ParB-HT complex and the hyphal tip of the wild type control (strain KP006) and the *smc* strain (KP007) (Fig. 4A**)**. Additionally, we analysed the positions of FROS-marked chromosomal regions located at three different distances from *oriC*: 52 kb, 112 kb, and 353 kb in both the wild type (KP012, KP013, KP014) and *smc* backgrounds (KP015, KP016, KP017), measuring their distances to the hyphal tip (Fig. 4B**)**.

In the wild type background, the ParB-HT complex was positioned approximately 1.09 ± 0.03 μm from the hyphal tip. In contrast, in the *smc* background, the ParB-HT complex was shifted away from the tip and positioned at 1.23 ± 0.05 μm (p-value = 5.09e-05, two-tailed Student’s t-test) (Fig. 4C). Similarly, the distance between the hyphal tip and the FROS-marked *oriC* (FROS-52 signal) increased in the absence of SMC, measuring 1.81 ± 0.07 μm in *smc* compared to 1.62 ± 0.08 μm in the wild type (p-value = 0.0031, Wilcoxon test two-sided) (Fig. 4D). Notably, the positions of the FROS-marked regions 112 kb and 353 kb away from *oriC* were only marginally or not affected by the *smc* deletion (p-values = 0.03 and 1, respectively) (Fig. 4D). These results suggest that the absence of SMC increases the distance between the hyphal tip and *oriC*, but does not affect the positioning of *oriC*-distal chromosomal regions, indicating that SMC contributes to the organization and anchoring the *oriC* region.

To further understand how SMC influences the positioning of *oriC* within the nucleoid, we measured the distance between the ParB-HT complex or the FROS signal and the boundary of the Hoechst-stained nucleoid (Fig. 4E, F). In the control strain, the ParB-HT complex was localized precisely the tip-proximal boundary of the nucleoid, at a distance of 0.17 ± 0.04 μm (Fig. 4G). In the *smc* strain, however, the distance between the ParB-HT complex and the edge of the nucleoid increased significantly, averaging 0.45 ± 0.06 μm (p-value = 2.2e-16, Wilcoxon test two-sided) (Fig. 4G**)**. Similarly, the FROS-52 signal, which is located 52 kb from *oriC*, was close to the boundary of the nucleoid in the wild type background (0.71 ± 0.07 μm) (Fig. 4H**)**. However, in the absence of SMC, the FROS-52 signal shifted away from the edge of the nucleoid to 1.2 ± 0.11 μm (p-value = 8.6e-10, Wilcoxon two-sided), and the variation of its position also increased (Fig. 4H**)**. Interestingly, the positions of the two other FROS-marked chromosomal regions—120 kb and 353 kb from *oriC*—were not significantly affected by the *smc* deletion, with p-values of 0.16 and 0.06, respectively (Fig. 4H**)**. This reinforces the notion that the positioning of these regions does not depend on SMC. In conclusion, our measurements demonstrate that SMC-dependent chromosome organization is crucial for maintaining the proximity of *oriC* to the cell tip and its localization at the edge of the nucleoid. This suggests that SMC plays a significant role in organizing the *oriC* region but does not influence the positioning of chromosomal regions located further from *oriC*.

## Discussion

 SMC was earlier established to play a crucial role in ensuring the efficient compaction of *Streptomyces venezuelae* chromosomes within spores and in aligning the chromosomal arm﻿s^46,47,67^. Consequently, we hypothesized that disrupting DNA organization by removing SMC would negatively impact spore germination. Our studies presented here demonstrate that SMC elimination interferes with chromosome replication during germination and the early growth. We also show that the lack of SMC delays the targeting of chromosome to the germ tube. Furthermore, the absence of SMC mispositions the *oriC* region within the nucleoid and in relation to the cell pole, while the locations of chromosomal regions distal from *oriC* remain largely unaffected.

During *Streptomyces* spore germination, DNA replication precedes germ tube emergence and delivers chromosomal copies which populate the forming germ tube. The loss of SMC significantly increased the *ori/arm* ratio during germination, suggesting multiple events of chromosomal replication initiation. While a slight induction of sporulation partially explains the increased *ori/arm* ratio, the elevated number of *oriC*s likely results from either over-initiation of replication or a reduced speed of replication forks. We lean towards the latter explanation, given that during germ tube extension, we observed somewhat higher density of the *oriC*s (ParB complexes) in the strain lacking SMC as compared to the wild type control, but density of replisomes was similar. Additionally, chromosomal copies multiplied slower in extending hyphae of strain lacking SMC and we detected modified patterns of transcriptional activity of the selected promoters. Surprisingly, even though germ tubes emerged efficiently, their population with the chromosome and subsequent elongation were delayed in the absence of SMC. Our earlier studies demonstrated that germ tube or branch extension is inhibited in the absence of chromosomal DNA^56^; however, the exact mechanism of this checkpoint remains unknown. We infer that inefficient chromosome replication, may be, at least partially, responsible for the delayed targeting of the chromosome to the germ tube.

Interestingly, earlier studies showed that the lack of SMC did not affect DNA replication or growth in later stages of *S. venezuelae* development^62^. This raises the question: why does SMC-dependent chromosome organization only impact the replication rate during the early growth stages? One plausible explanation is that replication is more intense at this stage. In contrast, during later growth stages, only a few of multiple chromosomal copies undergo replication, thus the impact of SMC may be less pronounced^52^. Moreover, in late vegetative growth, chromosomes are primarily in an open conformation with limited interactions between their arms, suggesting limited loading of SMC^46^. Interestingly, studies on *Bacillus subtilis* sporulation did not show that inefficient loading of condensin affected replication, as determined by marker frequency analysis^68^. In those studies, condensin loading was completely abolished only during germination, whereas we analysed the germination of spores lacking SMC and with altered chromosome arrangements. More recent research indicates that inactivation of condensin in *B. subtilis* led to the interlinking of sister chromosomes under rapid growth conditions. However, slowing the replication rate was sufficient to resolve this issue. Notably, recent studies of *C. crescentus* revealed that replisome movement relies on SMC^31^. In *M. smegmatis*, one of the condensin homologues, known as Mks, was found to form clusters closely associated with replisomes. This may suggest its involvement in the organization of newly replicated DNA^69^. Our findings demonstrate a critical role for SMC in DNA replication at the very early stages of the *S. venezuelae* life cycle, when this process is most active. These findings also align with the influence of SMC on replisome progression and sister-origin separation described in *B. subtilis* and *C. crescentus*^31,68,70^.

**Fig. 5:**
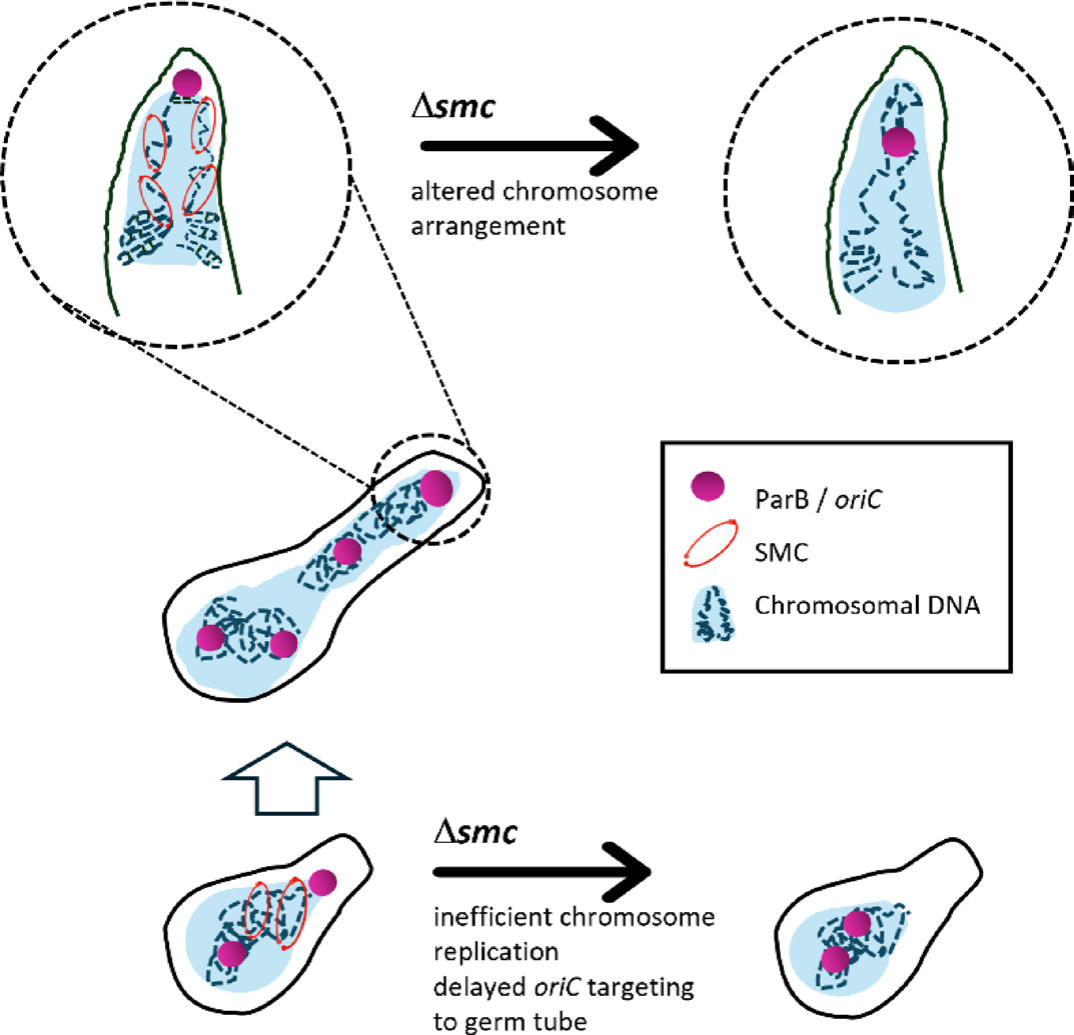
Model of SMC impact on* Streptomyces* spore germination and* oriC* position at the apical boundary of the nucleoid. SMC positions *oriC* at the boundary of the nucleoid, imposing *ori-ter* organization and allowing efficient progress of replication and chromosome targeting to the germ tube during spore germination. The absence of SMC disturbs *ori-ter* organization of the apical chromosome, impairs progress of DNA replication, and interferes with its targeting to a germ tube.

The key finding of our studies is that SMC contributes to organization of the apical chromosome during the extension of hyphal cells. We conclude that SMC organizes the chromosomal region near *oriC*, positioning the ParB complex at the apical edge of the nucleoid. This organization imposes an *ori-ter* chromosome orientation and facilitates the anchoring of *oriC* at the tip of the hypha. However, several questions remain unanswered: Do all chromosomes exhibit the same *ori-ter* organization, and is SMC loaded with the same efficiency across all chromosomal copies in the young hyphal cell? We previously showed that apical *oriC* anchorage facilitates targeting of the chromosome into the germ tube during germination. Our earlier studies demonstrated that the precise positioning of *oriC* at the apical edge of the nucleoid is mediated by the ParB complex, which interacts with ParA localized at the tip of the hyphal cell^56^. This polar recruitment of ParA depends on its interaction with Scy, a component of the apical protein complex known as the polarisome (or TIPOC)^56,57^. Therefore, the observed disturbed nucleoid architecture with mislocalised *oriC* could explain the delayed targeting of the chromosome to the germ tube. Interestingly, labelling of three independent chromosomal regions revealed that SMC controls the position of the region located 52 kb from *oriC*, while regions more distal to *oriC* were less precisely positioned within the cell and showed a lower dependency on SMC. The influence of SMC on the positioning of *oriC* relative to the nucleoid and the cell pole supports the notion that the SMC-dependent organization of the chromosome facilitates targeting the chromosome to the germ tube (Fig. 5**)**.

Studies on other bacterial species have shown that condensins play a crucial role in the segregation and positioning of the *oriC* in *B. subtilis* and *E. coli*^42,43,70,71^. In widened *B. subtilis* cells, *oriC* is located at the end of the crescent shape formed by the nucleoid^71^. This shape is maintained by SMC, which accumulates near the *oriC* region of the chromosome. These findings align with data from chromosome conformation capture, which demonstrated that SMC induces alignment of the chromosomal arms^28,29^. Tišma and colleagues noted that chromosomal DNA is more condensed near the *oriC* region, suggesting that this arrangement is influenced by the targeted loading of SMC facilitated by ParB^71^. This supports the notion that chromosome structure determines the localization of specific chromosomal regions within the cell^72^. Thus, our discovery that SMC positions *oriC* within *Streptomyces* hyphal cells is consistent with earlier observations in model bacteria. We build on prior studies by demonstrating that the influence of SMC on chromosomal DNA location is dependent on the distance from the *oriC* region.

In summary, we demonstrate that abnormal chromosome organization in germinating spores lacking SMC impairs replication and delays the targeting of chromosomes to the germ tube. Additionally, we show that SMC helps position the *oriC* region at the boundary of the apical chromosome. We infer that SMC facilitates efficient replication by organizing the chromosomal DNA, in the proximity to *oriC*. Moreover, we postulate that SMC-dependent organization of the *oriC*-proximal region of the chromosome is not only critical for replication progress but also positions oriC for anchorage at the hyphal cell pole.

## Materials and methods

### Bacterial strains and culture conditions

The constructs used in this study are listed in Table S1, while the bacterial strains utilised are detailed in Table S2. Genetic modifications were carried out following standard protocols and in accordance with the guidelines provided by the specific enzyme or kit manufacturers. Oligonucleotide synthesis and sequencing reactions were performed by Genomed (Poland), and Sanger sequencing was performed by Microsynth (Switzerland). The culture conditions and handling methods for *E. coli* followed established procedures^73^. Detailed strategies for strain construction are provided in the Supplementary Information. The *S. venezuelae* strains used in this research are derivatives of the NRRL B-65,442 strain, recognised as the wild type *S. venezuelae* strains were grown on maltose yeast extract medium (MYM), R2 solid medium without sucrose (R2/S) agar plates, or in MYM liquid medium, with the addition of appropriate antibiotics, as previously described^74^. For all experiments, the volume of spore suspension added to the medium was calculated based on the optical density (OD_600_) measured for a diluted spore suspension in 10% glycerol. After diluting the spore suspension to an OD_600_ of 0.05, 1 µL was used to inoculate 5 mL of MYM liquid medium. The germination efficiency was measured by inoculation of MYM liquid medium with the same amount of spores (determined by OD_600_ measurement) and microscopical determination of the fraction of germinating spores after the specific time of culture incubation. The growth rate of *S. venezuelae* was analysed according to previously described protocols (58). The cultures for bioluminescence analyses are described in Supplementary Information. An intergeneric conjugation procedure was used to introduce cosmid or plasmid DNA into *S. venezuelae* using an *E. coli* ET12567/pUZ8002 strain, which contained a conjugative plasmid (pUZ8002). Conjugation was performed using established methods, and exconjugants were selected on MYM medium^58^. The *S. venezuelae* spores were stored at -70 °C.

### Microscopy and image analyses

For the preparation of microscopic specimens, *S. venezuelae strains* were grown in 2 ml of liquid MYM medium at 30 °C. The fluorescent dyes - Hoechst 33,342 for DNA staining or ligands for the HaloTag protein - TMR *direct ligand* or Janelia Fluor 549 - were added to the medium immediately after inoculation with the appropriate amount of spores. For slides preparation, the cultures were washed three times with 2 ml of PBS buffer (centrifugation 2 min, 1800 rpm, 4 °C) and resuspended in a volume of 50–200 µl of PBS buffer (depending on the amount of pellet). 2 µl culture suspension was placed on microscopic slides (PTFE slide with Teflon coating; Epredia - B-0507) covered with 1.2% agarose in milliQ water. After drying (15 min), the specimen was pressed with a 24 × 24 mm cover slip.

Analyses of the microscope samples were performed using the Leica LAS X Widefield Systems fluorescence microscope equipped with Leica HC PL Fluotar 100x/1.32 OIL PH3 lens, filter Set: DAPI ET, TXR ET, Y3 ET, GFP ET, CFP ET, YFP ET, and Leica DFC7000 GT digital camera and software platform - LAS X. The exposure time for TetR-mVenus was 200 ms (YFP filter), DnaN-EGFP 180 ms (GFP filter) for HaloTag TMR *direct ligand* (Promega) or Janelia Fluor-549 (Promega) − 200 ms (mCherry filter) and for Hoechst 33,342 (trihydrochloride trihydrate) (Invitrogen) 100 ms (DAPI filter). The detection of the fluorescent signal was performed using the ImageJ program (https://fiji.sc/) and script “*findpeaks*” available on the *Microbes in Wrocław* Shiny server (http://microbesinwroclaw.biotech.uwr.edu.pl:3838/zmm_apps/*).* Fluorescence profiles were determined using the ImageJ (“*Straight segmented or freehand lines*,* or arrows*”) linear selection tool with an appropriately selected width. Distances between the fluorescent signals and the selected reference points were measured using a “*findpeaks”* that locates fluorescent complexes in hyphae via the R Peaks package. Each biological repetition was performed in two technical repetitions. The results were then averaged, and statistical significance was analysed.

To carry out time-lapse analyses, the DeltaVision Elite Microscope (GE Healthcare), equipped with the Olympus PLANApo 100x/1.40 OIL PH3 lens, FITC, YFP, and mCherry filters, and the CellAsic Onix microfluidic system (Milipore), was used. *S. venezuelae* cultures were prepared based on a published protocol^75^. For the observation, *S. venezuelae* spores were loaded onto a microfluidics plate (B04A, Milipore) in the observation chamber at a pressure of 6 PSI for 2–15 s. After loading the spore, the experiment was conducted at a constant pressure of 3 PSI and a temperature of 30 °C. In each experiment, fresh MYM medium was first collected from well no. 1 for the first 6 h. Thirty minutes after loading the spores into the observation chamber, preliminary observations were made, and the positions for the analyses were marked (softWoRx software). After determining the position (30 min to 2 h after loading the cells), the analysis was initiated and conducted for 1–6 h, with pictures taken at the selected positions every 10 min. To observe DnaN-EGFP using the FITC filter, a 200-ms exposure time at 10% transmission was used. For TetR-mVenus YFP filter, 200 ms exposure time at 32% transmission, and for HaloTag ligand Janelia Fluor-549 mCherry filter with 200 ms exposure at 30% transmission. Data were collected from two biological replicates. Each biological repetition was performed in two technical repetitions.

Analyses of time-lapse movies were performed using the ImageJ program and the “*findpeaks*” script available on the Microbes in Wrocław Shiny server (http://microbesinwroclaw.biotech.uwr.edu.pl:3838/zmm_apps/findpeaks/*).* This script was used for cell length analysis, analysis of fluorescent cluster position, and analysis of the timing of fluorescence signal appearance (ParB-HaloTag, DnaN-EGFP). The “*Straight segmented or freehand lines*,* or arrows”* tool in ImageJ, with appropriately selected widths, was used to locate local maxima in fluorescence profiles on microscopic images and to determine the rate of hyphal elongation (Δl/Δt). Then, the results were averaged, and 95% confidence intervals were calculated for each mean; statistical significance was analysed. Obtained p-values were corrected for multiple comparisons using Holm’s method.

### Marker frequency analysis

Marker frequency analysis, used to determine the *oriC/arm* ratio, was performed using quantitative PCR (qPCR) with *S. venezuelae* chromosomal DNA as the template. Cultures for DNA isolation were carried out in 5 mL of MYM medium, inoculated with 1 µL of spore suspension with an OD600 of 0.05. Cultures were carried out in plastic 50 ml Falcon tubes for 2 to 28 h at 30 °C, with shaking (200 rpm). Chromosomal DNA was isolated using the protocol described earlier^75^. After isolation, the DNA concentration was measured spectrophotometrically using the NanoDrop 1000, and then the DNA was diluted to a final concentration of 1 ng/µl. An 83 bp DNA fragment near the *oriC* region (location on *S. venezuelae* chromosome 3,958,240–3,958,322 bp) was amplified with primers: gyr2_Fw/gyr2_Rv, and a 78 bp DNA fragment located at the end of the right arm of the chromosome (position on S. *venezuelae* chromosome: 1,320,274–1,320,351) was amplified with primers: arg3_Fw/arg3_Rv (Table S3). qPCR was performed using *PowerUp SYBR Green Master Mix* on the StepOnePlus apparatus (Applied Biosystems). The *oriC/arm* ratio was calculated using the comparative ΔΔCt method. The reference sample was *the oriC*/*arm* ratio, estimated at 1 in a DNA sample from a culture grown for 26 h, corresponding to the appearance of spores, i.e., the stage at which chromosome replication is stopped.

## Supplementary Information

Below is the link to the electronic supplementary material.

Supplementary Material 1

Supplementary Material 2

## Data Availability

The raw data are available at https://doi.org/10.34616/UDJ363.

## References

[1] Badrinarayanan, A., Le, T. B. K. & Laub, M. T. Bacterial chromosome organization and segregation. *Annu. Rev. Cell. Dev. Biol.* **31**, 171–199 (2015). 26566111 10.1146/annurev-cellbio-100814-125211 PMC4706359

[2] Ponndara, S., Kortebi, M., Boccard, F., Bury-Moné, S. & Lioy, V. S. Principles of bacterial genome organization, a conformational point of view. *Mol. Microbiol.* **123**, 195–205 (2025). 38922728 10.1111/mmi.15290 PMC11894783

[3] Harju, J. & Broedersz, C. P. Physical models of bacterial chromosomes. *Mol. Microbiol.* **123**, 143–153 (2025). 38578226 10.1111/mmi.15257 PMC11841833

[4] Wang, X., Montero Llopis, P. & Rudner, D. Z. Organization and segregation of bacterial chromosomes. *Nat. Rev. Genet.* **14**, 191–203 (2013). 23400100 10.1038/nrg3375 PMC3869393

[5] Le Berre, D., Reverchon, S., Muskhelishvili, G. & Nasser, W. Relationship between the chromosome structural dynamics and gene expression—A chicken and Egg Dilemma? *Microorganisms* 10 (2022). 10.3390/microorganisms10050846 PMC9144111 35630292

[6] Surovtsev, I. V. & Jacobs-Wagner, C. Subcellular organization: A critical feature of bacterial cell replication. *Cell* **172**, 1271–1293 (2018). 29522747 10.1016/j.cell.2018.01.014 PMC5870143

[7] Duigou, S. & Boccard, F. Long range chromosome organization in Escherichia coli: The position of the replication origin defines the non-structured regions and the Right and Left macrodomains. *PLoS Genet* **13**, (2017). 10.1371/journal.pgen.1006758 PMC5441646 28486476

[8] David, A. et al. The Two Cis-Acting Sites, parS1 and oriC1, Contribute to the longitudinal organisation of Vibrio cholerae Chromosome I. *PLoS Genet* **10**, (2014). 10.1371/journal.pgen.1004448 PMC4091711 25010199

[9] Umbarger, M. A. et al. The three-dimensional architecture of a bacterial genome and its alteration by genetic perturbation. *Mol. Cell.* **44**, 252–264 (2011). 22017872 10.1016/j.molcel.2011.09.010 PMC3874842

[10] Nielsen, H. J., Ottesen, J. R., Youngren, B., Austin, S. J. & Hansen, F. G. The Escherichia coli chromosome is organized with the left and right chromosome arms in separate cell halves. *Mol. Microbiol.* **62**, 331–338 (2006). 17020576 10.1111/j.1365-2958.2006.05346.x

[11] Yamaichi, Y. et al. A multidomain hub anchors the chromosome segregation and chemotactic machinery to the bacterial pole. *Genes Dev.* **26**, 2348–2360 (2012). 23070816 10.1101/gad.199869.112 PMC3475806

[12] Holówka, J., Trojanowski, D., Janczak, M., Jakimowicz, D. & Zakrzewska-Czerwińska, J. The origin of chromosomal replication is asymmetrically positioned on the mycobacterial nucleoid, and the timing of its firing depends on HupB. *J. Bacteriol* **200**, (2018). 10.1128/JB.00044-18 PMC5915789 29531181

[13] Lin, L., Valeriano, O., Harms, M., Søgaard-Andersen, A., Thanbichler, M. & L. & Bactofilin-mediated organization of the ParABS chromosome segregation system in Myxococcus xanthus. *Nat. Commun.* **8**, 1817 (2017). 29180656 10.1038/s41467-017-02015-z PMC5703909

[14] Bowman, G. R. et al. A Polymeric protein anchors the chromosomal Origin/ParB complex at a bacterial cell pole. *Cell* **134**, 945–955 (2008). 18805088 10.1016/j.cell.2008.07.015 PMC2745220

[15] Harms, A., Treuner-Lange, A. & Schumacher, D. Søgaard-Andersen, L. Tracking of chromosome and replisome dynamics in Myxococcus xanthus reveals a novel chromosome arrangement. *PLoS Genet.* **9**, e1003802–e1003802 (2013). 24068967 10.1371/journal.pgen.1003802 PMC3778016

[16] Youngren, B., Nielsen, H. J., Jun, S. & Austin, S. The multifork Escherichia coli chromosome is a self-duplicating and self-segregating thermodynamic ring polymer. *Genes Dev.* **28**, 71–84 (2014). 24395248 10.1101/gad.231050.113 PMC3894414

[17] Wang, X., Montero Llopis, P. & Rudner, D. Z. Bacillus subtilis chromosome organization oscillates between two distinct patterns. *Proc. Natl. Acad. Sci.* **111**, 12877–12882 (2014). 25071173 10.1073/pnas.1407461111 PMC4156703

[18] Le, T. B. K., Imakaev, M. V., Mirny, L. A. & Laub, M. T. High-resolution mapping of the spatial organization of a bacterial chromosome. *Sci. (1979)*. **342**, 731–734 (2013). 10.1126/science.1242059 PMC3927313 24158908

[19] Mäkelä, J. & Sherratt, D. SMC complexes organize the bacterial chromosome by lengthwise compaction. *Curr. Genet.* **66**, 895–899 (2020). 32300862 10.1007/s00294-020-01076-w PMC7497336

[20] Uhlmann, F. A unified model for cohesin function in sister chromatid cohesion and chromatin loop formation. *Mol. Cell*. **85**, 1058–1071 (2025). 40118039 10.1016/j.molcel.2025.02.005

[21] Nolivos, S. & Sherratt, D. The bacterial chromosome: architecture and action of bacterial SMC and SMC-like complexes. *FEMS Microbiol. Rev.* **38**, 380–392 (2014). 24118085 10.1111/1574-6976.12045 PMC4255302

[22] Yamazoe, M. et al. Complex formation of MukB, MukE and MukF proteins involved in chromosome partitioning in Escherichia coli. *EMBO J.* **18**, 5873–5884 (1999). 10545099 10.1093/emboj/18.21.5873 PMC1171653

[23] Diebold-Durand, M. L. et al. Structure of full-length SMC and rearrangements required for chromosome organization. *Mol. Cell.* **67**, 334–347e5 (2017). 28689660 10.1016/j.molcel.2017.06.010 PMC5526789

[24] Bürmann, F. et al. Tuned SMC arms drive chromosomal loading of prokaryotic condensin. *Mol. Cell.* **65**, 861–872e9 (2017). 28238653 10.1016/j.molcel.2017.01.026 PMC5344682

[25] Hirano, T. & Kinoshita, K. SMC-mediated chromosome organization: Does loop extrusion explain it all? *Current Opin. Cell. Biology* **92** (2025). 10.1016/j.ceb.2024.102447 39603149

[26] Marko, J. F., De Los Rios, P., Barducci, A. & Gruber, S. DNA-segment-capture model for loop extrusion by structural maintenance of chromosome (SMC) protein complexes. *Nucleic Acids Res.* **47**, 6956–6972 (2019). 31175837 10.1093/nar/gkz497 PMC6649773

[27] Ganji, M. et al. Real-time imaging of DNA loop extrusion by condensin. *Science* **360**, 102–105 (2018). 29472443 10.1126/science.aar7831 PMC6329450

[28] Wang, X. et al. Condensin promotes the juxtaposition of DNA flanking its loading site in *Bacillus subtilis*. *Genes Dev.* **29**, 1661–1675 (2015). 26253537 10.1101/gad.265876.115 PMC4536313

[29] Wang, X., Brandao, H. B., Le, T. B. K., Laub, M. T. & Rudner D. Z. Bacillus subtilis SMC complexes juxtapose chromosome arms as they travel from origin to terminus. *Science* **355**, 524–527 (2017). 28154080 10.1126/science.aai8982 PMC5484144

[30] Hiraga, S. et al. Mutants defective in chromosome partitioning in* E. coli.**Res. Microbiol.* **142**, 189–194 (1991). 1925018 10.1016/0923-2508(91)90029-a

[31] Zhang, C. et al. Chromosome organization shapes replisome dynamics in *Caulobacter crescentus*. *Nat Commun* **15**, (2024). 10.1038/s41467-024-47849-6 PMC11043382 38658616

[32] Gruber, S. & Errington, J. Recruitment of condensin to replication origin regions by ParB/SpoOJ promotes chromosome segregation in* B. subtilis*. *Cell* **137**, 685–696 (2009). 19450516 10.1016/j.cell.2009.02.035

[33] Sullivan, N. L., Marquis, K. & Rudner, D. Z. Recruitment of SMC by ParB-parS organizes the origin region and promotes efficient chromosome segregation. *Cell* **137**, 697–707 (2009). 19450517 10.1016/j.cell.2009.04.044 PMC2892783

[34] Tišma, M., Kaljević, J., Gruber, S., Le, T. B. K. & Dekker, C. Connecting the dots: key insights on ParB for chromosome segregation from single-molecule studies. *FEMS Microbiol. Reviews* **48** (2024). 10.1093/femsre/fuad067 PMC10786196 38142222

[35] Pióro, M. & Jakimowicz, D. Chromosome segregation proteins as coordinators of cell cycle in response to environmental conditions. *Front. Microbiol.* **11**, 588 (2020). 32351468 10.3389/fmicb.2020.00588 PMC7174722

[36] Kawalek, A., Wawrzyniak, P., Bartosik, A. A. & Jagura-Burdzy, G. Rules and exceptions: The role of chromosomal ParB in DNA segregation and other cellular processes. *Microorganisms* **8**, 10–12 (2020). 10.3390/microorganisms8010105 PMC7022226 31940850

[37] Lutkenhaus, J. The ParA / MinD family puts things in their place. *Trends Microbiol.* **20**, 411–418 (2012). 22672910 10.1016/j.tim.2012.05.002 PMC3436946

[38] Zhang, H. & Schumacher, M. A. Structures of partition protein ParA with nonspecific DNA and ParB effector reveal molecular insights into principles governing Walker-box DNA segregation. *Genes Dev.* **31**, 1–12 (2017). 28373206 10.1101/gad.296319.117 PMC5393062

[39] Jalal, A. S. B. & Le, T. B. K. Bacterial chromosome segregation by the ParABS system. *Open. Biol.* **10**, 200097 (2020). 32543349 10.1098/rsob.200097 PMC7333895

[40] Jalal, A. S. et al. A CTP-dependent gating mechanism enables ParB spreading on DNA. *Elife* (2021). 10.7554/eLife.69676 10.7554/eLife.69676 PMC8367383 34397383

[41] Graham, T. G. W. et al. ParB spreading requires DNA bridging. *Genes Dev.* **28**, 1228–1238 (2014). 24829297 10.1101/gad.242206.114 PMC4052768

[42] Gruber, S. Multilayer chromosome organization through DNA bending, bridging and extrusion. *Curr. Opin. Microbiol.* **22**, 102–110 (2014). 25460803 10.1016/j.mib.2014.09.018

[43] Badrinarayanan, A., Lesterlin, C., Reyes-Lamothe, R. & Sherratt, D. The escherichia coli SMC complex, MukBEF, shapes nucleoid organization independently of DNA replication. *J. Bacteriol.* **194**, 4669–4676 (2012). 22753058 10.1128/JB.00957-12 PMC3415497

[44] Danilova, O., Reyes-Lamothe, R., Pinskaya, M., Sherratt, D. & Possoz, C. MukB colocalizes with the oriC region and is required for organization of the two Escherichia coli chromosome arms into separate cell halves. *Mol. Microbiol.* **65**, 1485–1492 (2007). 17824928 10.1111/j.1365-2958.2007.05881.x PMC2169520

[45] Szafran, M. J., Jakimowicz, D. & Elliot, M. A. Compaction and control-the role of chromosome-organizing proteins in Streptomyces. *FEMS Microbiol. Rev.* **44**, 725–739 (2020). 32658291 10.1093/femsre/fuaa028 PMC7685783

[46] Szafran, M. J. et al. Spatial rearrangement of the Streptomyces venezuelae linear chromosome during sporogenic development. *Nat. Commun.* **12**, 5222 (2021). 34471115 10.1038/s41467-021-25461-2 PMC8410768

[47] Kois, A. et al. SMC protein-dependent chromosome condensation during aerial hyphal development in Streptomyces. *J. Bacteriol.* **191**, 310–319 (2009). 18931116 10.1128/JB.00513-08 PMC2612454

[48] Flärdh, K., Richards, D. M., Hempel, A. M., Howard, M. & Buttner, M. J. Regulation of apical growth and hyphal branching in Streptomyces. *Curr. Opin. Microbiol.* **15**, 737–743 (2012). 23153774 10.1016/j.mib.2012.10.012

[49] Bobek, J., Šmídová, K. & Čihák, M. A waking review: Old and novel insights into the spore germination in Streptomyces. *Front. Microbiol.* **8** (2017). 10.3389/fmicb.2017.02205 PMC5693915 29180988

[50] Flärdh, K. & Buttner, M. J. Streptomyces morphogenetics: dissecting differentiation in a filamentous bacterium. *Nat. Rev. Microbiol.* **7**, 36–49 (2009). 19079351 10.1038/nrmicro1968

[51] Chater, K. F. Recent advances in understanding streptomyces. *F1000Research* 5 (2016). 10.12688/f1000research.9534.1 PMC5133688 27990276

[52] Ruban-Ośmiałowska, B., Jakimowicz, D., Smulczyk-Krawczyszyn, A. & Chater, K. F. K. F. Zakrzewska-Czerwińska, J. Replisome localization in vegetative and aerial hyphae of Streptomyces coelicolor. *J. Bacteriol.* **188**, 7311–7316 (2006). 17015671 10.1128/JB.00940-06 PMC1636232

[53] Wolański, M. et al. Replisome trafficking in growing vegetative hyphae of Streptomyces coelicolor A3(2). *J. Bacteriol.* **193**, (2011). 10.1128/JB.01326-10 PMC3067586 21193604

[54] Jakimowicz, D. & van Wezel, G. P. G. P. Cell division and DNA segregation in Streptomyces: how to build a septum in the middle of nowhere? *Mol. Microbiol.* **85**, 393–404 (2012). 22646484 10.1111/j.1365-2958.2012.08107.x

[55] Bush, M. J., Casu, B. & Schlimpert, S. Dividing lines: compartmentalisation and division in Streptomyces. *Current Opin. Microbiol.* **85** (2025). 10.1016/j.mib.2025.102611 40300397

[56] Kois-Ostrowska, A. et al. Unique function of the bacterial chromosome segregation machinery in apically growing streptomyces - targeting the chromosome to new hyphal tubes and its anchorage at the tips. *PLoS Genet.* **12**, e1006488–e1006488 (2016). 27977672 10.1371/journal.pgen.1006488 PMC5157956

[57] Ditkowski, B. et al. Dynamic interplay of ParA with the polarity protein, Scy, coordinates the growth with chromosome segregation in Streptomyces coelicolor. *Open. Biol.* **3**, 130006 (2013). 23536551 10.1098/rsob.130006 PMC3718342

[58] Donczew, M. et al. ParA and ParB coordinate chromosome segregation with cell elongation and division during Streptomyces sporulation. *Open. Biol.* **6**, 150263 (2016). 27248800 10.1098/rsob.150263 PMC4852455

[59] Bury-Moné, S., Thibessard, A., Lioy, V. S. & Leblond, P. Dynamics of the streptomyces chromosome: chance and necessity. *Trends Genet.* **39**, 873–887 (2023). 37679290 10.1016/j.tig.2023.07.008

[60] Lioy, V. S. et al. Dynamics of the compartmentalized streptomyces chromosome during metabolic differentiation. *Nat. Commun.* **12**, 1–14 (2021). 34471117 10.1038/s41467-021-25462-1 PMC8410849

[61] Deng, L. et al. Dissection of 3D chromosome organization in Streptomyces coelicolor A3(2) leads to biosynthetic gene cluster overexpression. *Proc. Natl. Acad. Sci. U. S. A.* 120, (2023). 10.1073/pnas.2222045120 PMC10242723 36877856

[62] Pawlikiewicz, K. et al. SMC modulates ParB engagement in segregation complexes in streptomyces. *Nature Commun.* **16**, (2025). 10.1038/s41467-025-64044-3 PMC12511625 41068141

[63] Bobek, J., Strakova, E., Zikova, A. & Vohradsky, J. Changes in activity of metabolic and regulatory pathways during germination of S. coelicolor. *BMC Genomics* **15**, (2014). 10.1186/1471-2164-15-1173 PMC4367926 25539760

[64] Strakova, E. et al. Systems insight into the spore germination of streptomyces coelicolor. *J. Proteome Res.* **12**, 525–536 (2013). 23181467 10.1021/pr300980v

[65] Strakova, E., Bobek, J., Zikova, A. & Vohradsky, J. Global features of gene expression on the proteome and transcriptome levels in* S. coelicolor* during germination. *PLoS One* **8**, (2013). 10.1371/journal.pone.0072842 PMC3767685 24039809

[66] Strakova, E., Zikova, A. & Vohradsky, J. Inference of sigma factor controlled networks by using numerical modeling applied to microarray time series data of the germinating prokaryote. *Nucleic Acids Res.* **42**, 748–763 (2014). 24157841 10.1093/nar/gkt917 PMC3902916

[67] Dedrick, R. M., Wildschutte, H. & McCormick, J. R. Genetic interactions of smc, ftsK, and parB genes in Streptomyces coelicolor and their developmental genome segregation phenotypes. *J. Bacteriol.* **191**, 320–332 (2009). 18978061 10.1128/JB.00858-08 PMC2612423

[68] Gruber, S. et al. Interlinked sister chromosomes arise in the absence of condensin during fast replication in *B. subtilis*. *Curr. Biol.* **24**, 293–298 (2014). 24440399 10.1016/j.cub.2013.12.049 PMC3919155

[69] Bułacz, H., Hołówka, J. & Wójcik, W. & Zakrzewska-Czerwińska, J. MksB is a novel mycobacterial condensin that orchestrates spatiotemporal positioning of replication machinery. *Sci Rep* **14**, (2024). 10.1038/s41598-024-70054-w PMC11329512 39152186

[70] Wang, X., Tang, O. W., Riley, E. P. & Rudner, D. Z. The SMC condensin complex is required for origin segregation in* Bacillus subtilis*. *Curr. Biol.* **24**, 287–292 (2014). 24440393 10.1016/j.cub.2013.11.050 PMC3947903

[71] Tišma, M. et al. Direct observation of a crescent-shape chromosome in expanded* Bacillus subtilis* cells. *Nat. Commun.* **15**, (2024). 10.1038/s41467-024-47094-x PMC10979009 38548820

[72] Messelink, J. J. B., van Teeseling, M. C. F., Janssen, J. & Thanbichler, M. & Broedersz, C. P. Learning the distribution of single-cell chromosome conformations in bacteria reveals emergent order across genomic scales. *Nat. Commun.* **12**, (2021). 10.1038/s41467-021-22189-x PMC8010069 33785756

[73] Russell, D. W. & Sambrook, J. *Molecular Cloning: A Laboratory Manual*. *Cold Spring Harbour* (2001).

[74] Kieser, T., Bibb, M. J., Buttner, M. J., Chater, K. F. & Hopwood, D. A. Practical streptomyces genetics. *John Innes Centre Ltd.* **529**10.4016/28481.01 (2000).

[75] Schlimpert, S., Flärdh, K. & Buttner, M. Fluorescence time-lapse imaging of the complete *S. venezuelae* life cycle using a microfluidic device. *J. Vis. Exp.* **108**, (2016). 10.3791/53863 PMC4828195 26967231

