# Supplementary material for: SMC ensures efficient chromosome replication and oriC positioning during Streptomyces spore germination
Source: Sci Rep. 2026 Mar 15;16:13557. doi: 10.1038/s41598-026-43107-5 (PMC13121528; doi:10.1038/s41598-026-43107-5)
Supplement: Supplementary file 1 — Supplementary Material 1 [file 41598_2026_43107_MOESM1_ESM.pdf]

Supplementary Figures

SMC Ensures Efficient Chromosome Replication and *oriC* Positioning During *Streptomyces* Spore Germination

Katarzyna Pawlikiewicz[1], Agnieszka Strzałka[1], Agnieszka Nurek[1#], Magdalena Donczew[1&], Anna Gierlikowska[1&], Martyna Gongerowska-Jac[1], Marcin J. Szafran[1], Dagmara Jakimowicz[1*]

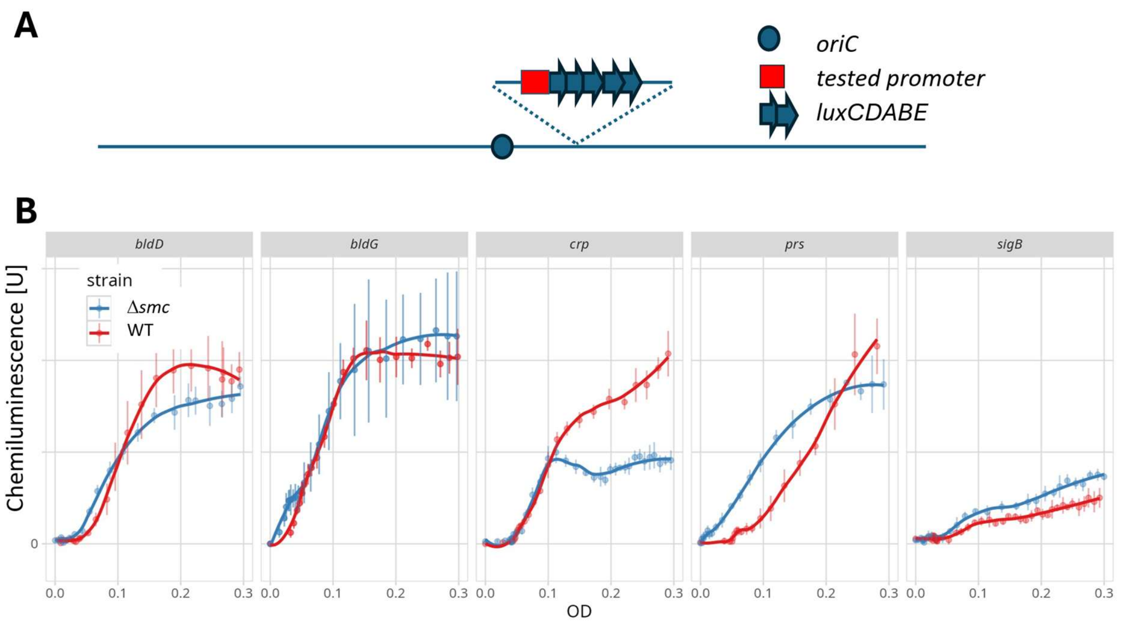

Fig. S1. The impact of *smc* deletion on transcriptional activity of selected promoters (*bldD, bldG, crp, prs, sigB*) determined by measurements of *lux* reporter genes activity. A. Scheme of the construct carrying reporter genes under the control of the tested promoters integrated in the *S. venezuelae* chromosome **B**. Chemiluminescence (U - arbitrary units) reflecting *lux* reporter genes activity during spore germination and early growth. Chemiluminescence analysis was performed using the Tecan Infinite 200 Pro plate reader, with measurements taken every 20 minutes. Error bars indicate the standard deviation. The data are derived from 5 experimental repeats.

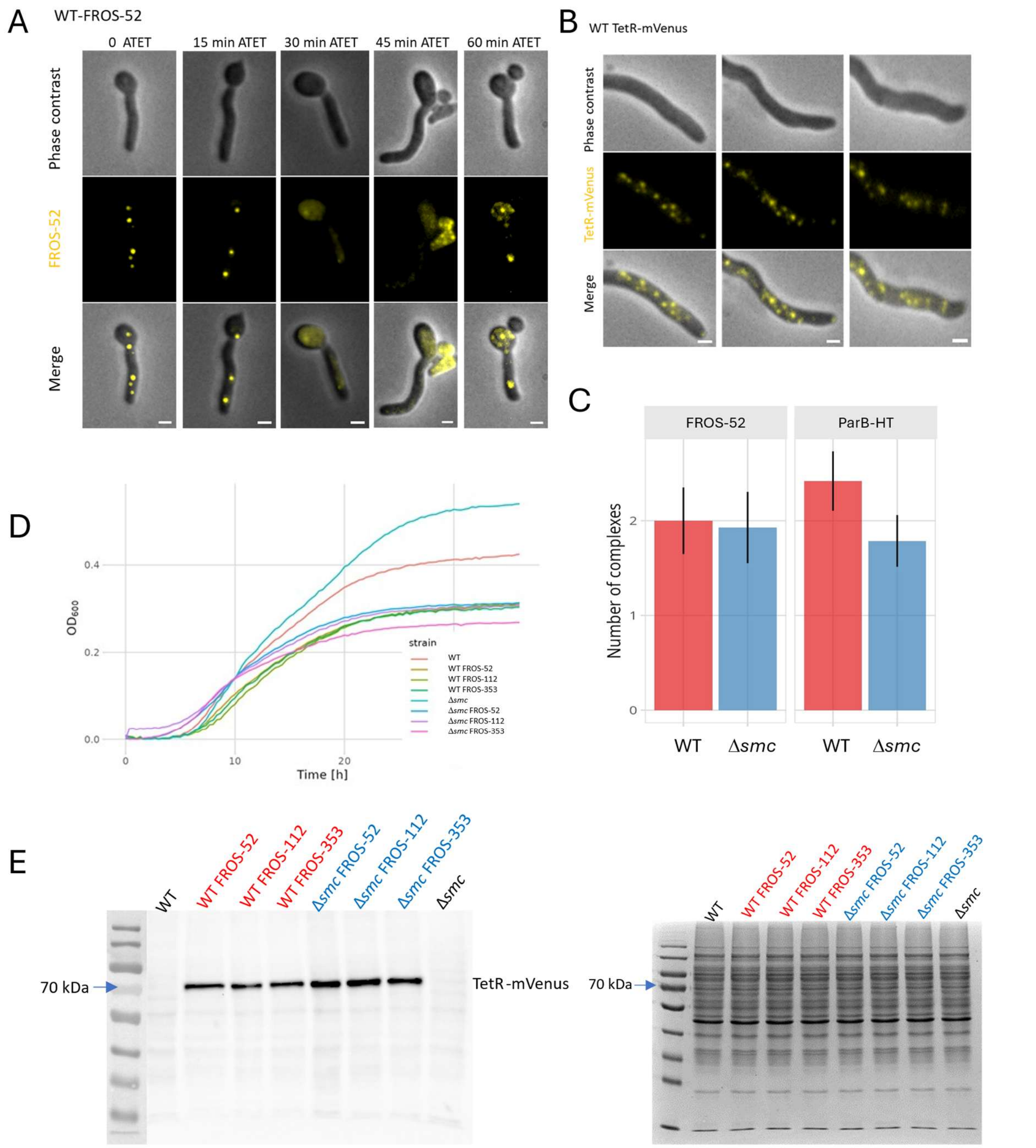

Fig. S2. Verification of *S. venezuelae* FROS strains. A. TetR-mVenus foci disassemble in the presence of anhydrotetracycline (ATET, 200 ng/ml)), confirming TetR-mVenus binding to DNA. Representative microscopy images showing the FROS-52 foci of TetR-mVenus in the wild type background (strain KP012) cultured for 5 hours in the absence of anhydrotetracycline (ATET), 15, 30, 45 and 60 min after addition of ATET. The bright field channel (grey, top panel), TetR-mVenus fluorescence (yellow, middle panel) and both channels overlaid (bottom panel) are shown. Scale bar – 1 μm. **B.** TetR-mVenus does not form distinct foci in the absence of *tetO*. Representative microscopy images showing the fluorescence of TetR-mVenus in the control strain (lacking *tetO,* strain KP021) cultured for 5 hours. **C.** The number of FROS-52 (TetR-mVenus) and ParB-HT complexes in hyphae after one hour of growth. The analyses were performed based on time-lapse analyses of the ParB-HT (KP006) and FROS-52 (KP012) strains. **D.** Growth analyses of FROS strains - the wild type: KP012 (WT, FROS-52), KP013 (WT,

FROS-112), KP014 (WT, FROS-353) and Δ*smc* background: KP015 (Δ*smc*, FROS-52), KP016 (Δ*smc*, FROS-112), KP017 (Δ*smc*, FROS-353). The growth rate was analysed using the OD_{600} measurement during cultures in Bioscreen C (three replicates for each culture). **E.** Analysis of TetR-mVenus levels in the wild type: KP012 (WT, FROS-52), KP013 (WT, FROS-112), KP014 (WT, FROS-353) and Δ*smc* background: KP015 (Δ*smc*, FROS-52), KP016 (Δ*smc*, FROS-112), KP017 (Δ*smc*, FROS-353). Left panel – representative Western blotting with anti-EGFP antibody, right panel – loading control (Coomassie blue-stained SDS-PAGE gel). Cell lysates containing 10 μg of protein were separated by SDS-PAGE in a 12.5% polyacrylamide gel.

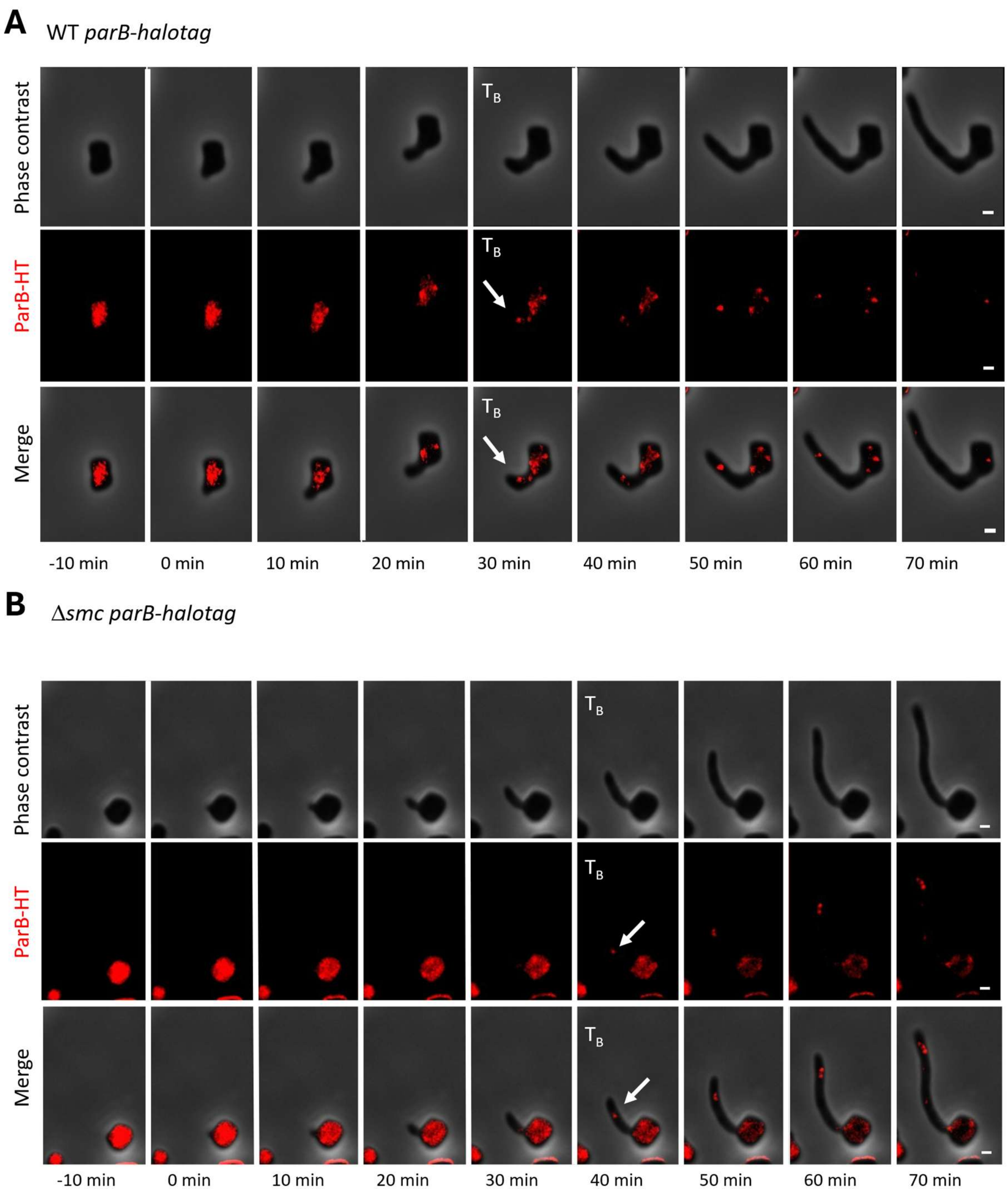

Fig S3. Microscopy analyses of ParB-HT focus appearance in the germ tube of the control and Δ*smc* strain. Representative images taken during time-lapse analyses of spore germination showing bright field images (grey, top panel), visualisation of ParB-HT stained with Janelia Fluor-549 (red, middle panel), and both channels overlaid (lower panel) in **A.** the wild type (KP006) and **B**. Δ*smc* background (KP007). 0 min - time of the germ tube emergence, time of ParB-HT focus appearance (T_B) in the germ tube is marked with the arrow. Scale bar: 1 µm.

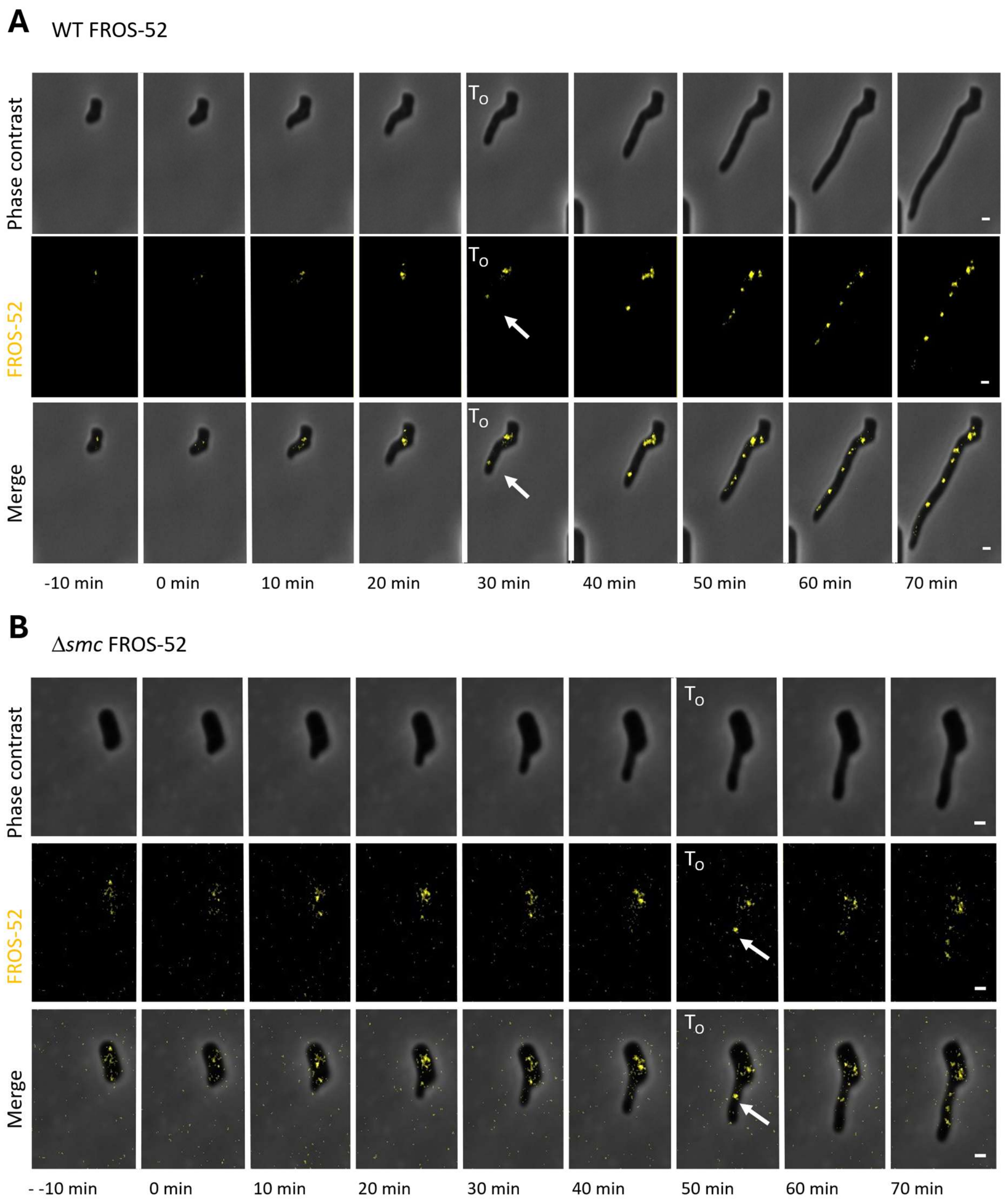

Fig. S4 Microscopy analyses of FROS-52 focus appearance in the germ tube of the control and Δ*smc* strain. Representative images taken during time-lapse analyses of spore germination showing bright field images (grey, top panel), TetR-mVenus fluorescence (yellow, middle panel) and both channels overlaid (bottom panel) in **A.** the wild type (KP012) and **B**. Δ*smc* background (KP015). 0 min - time of the germ tube emergence, time of FROS-52 focus appearance (T_O) in the germ tube is marked with the arrow. Scale bar: 1 µm.

A

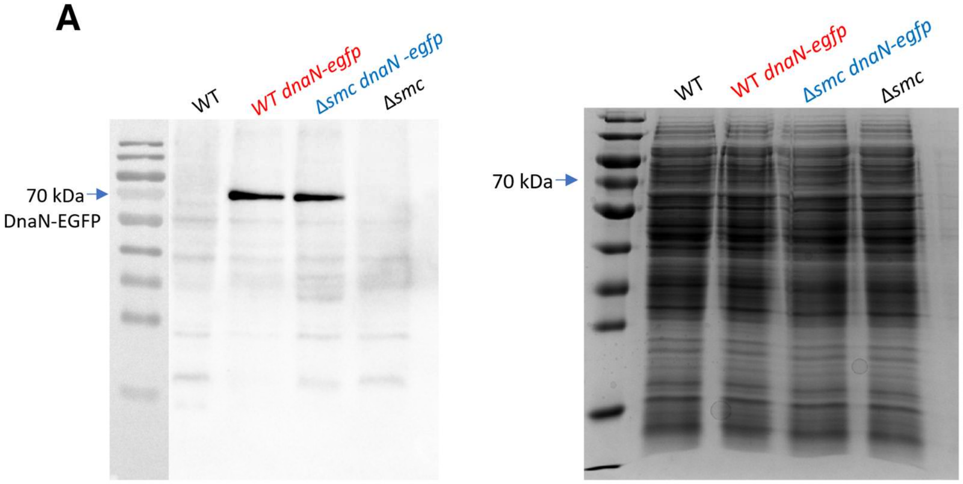

B

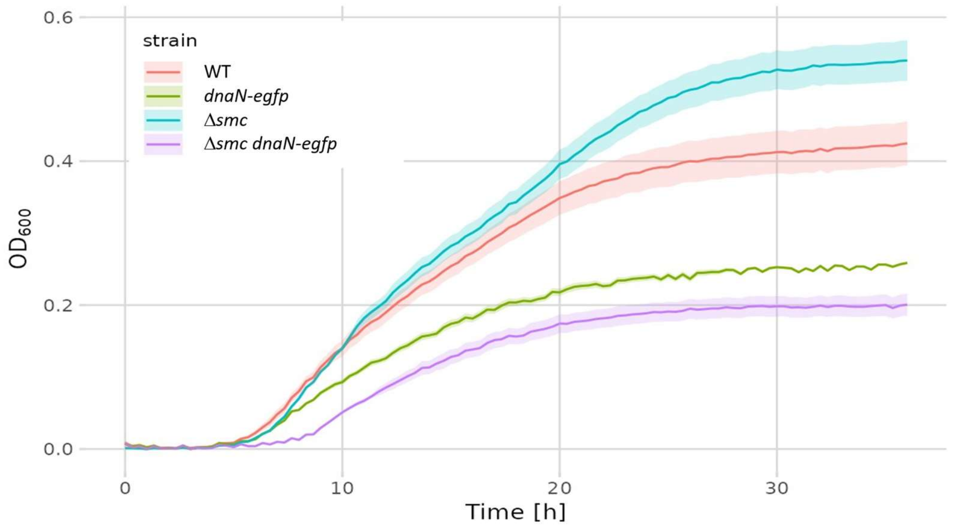

Fig. S5. Verification of DnaN-EGFP *S. venezuelae* strains. A Detection of DnaN-EGFP protein using the Western blotting with anti-EGFP antibodies in the wild type background (MD070, *dnaN-egfp*) and the Δ*smc* background (KPAG01 - Δ*smc dnaN-egfp*). Lysates derived from the wild-type strain (WT) and Δ*smc* (TM010) were used as controls. Cell lysates containing 10 µg of protein were separated by SDS-PAGE in a 12.5% polyacrylamide gel. **B**. Growth analyses of strains DnaN-EGFP producing in the wild type background (MD070, *dnaN-egfp*) and Δ*smc* background (KPAG01 - Δ*smc dnaN-egfp* as compared to wild-type strain (WT) and Δ*smc* (TM010). The growth rate was analysed using the OD_{600} measurement during cultures in Bioscreen C. Three replicates of each culture were analysed.

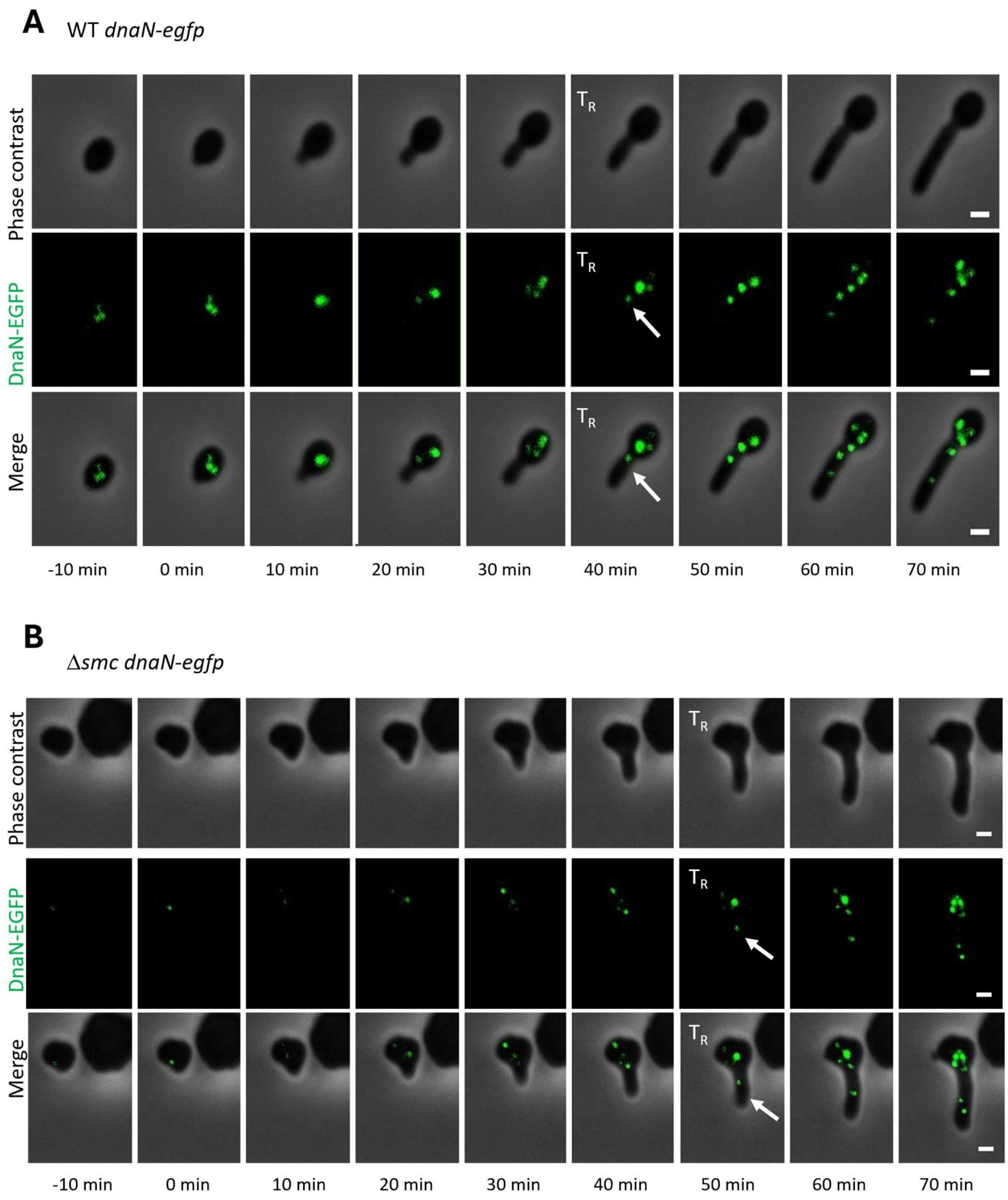

Fig. S6 Microscopy analyses of DnaN-EGFP focus appearance in the germ tube of the control and Δ*smc* strain. Representative images taken during time-lapse analyses of spore germination showing bright field images (grey, top panel), DnaN-EGFP fluorescence (green, middle panel), and both channels overlaid (bottom panel) overlaid on brightfield images (grey) in **A.** the wild type (MD070) and **B**. Δ*smc* background (KPAG01l). 0 min - time of the germ tube emergence, time of replisome appearance (T_R) in the germ tube is marked with the arrow. Scale bar: 1 µm.

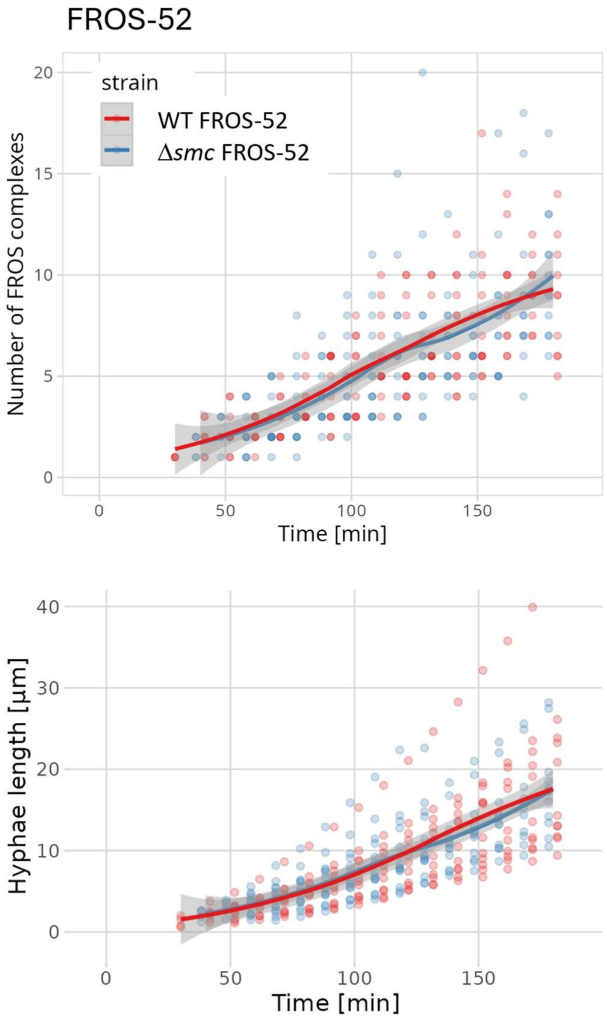

Fig. S7. The absence of SMC does not affect the number of FROS-52 complexes in the cell or the cell extension of the FROS-marked strain. A. The number of FROS-52 complexes in the vegetative cell, **B**. the length of the hyphal cell in the control strain (KP006) and Δ*smc* (KP007), counted during the time-lapse experiment**.**

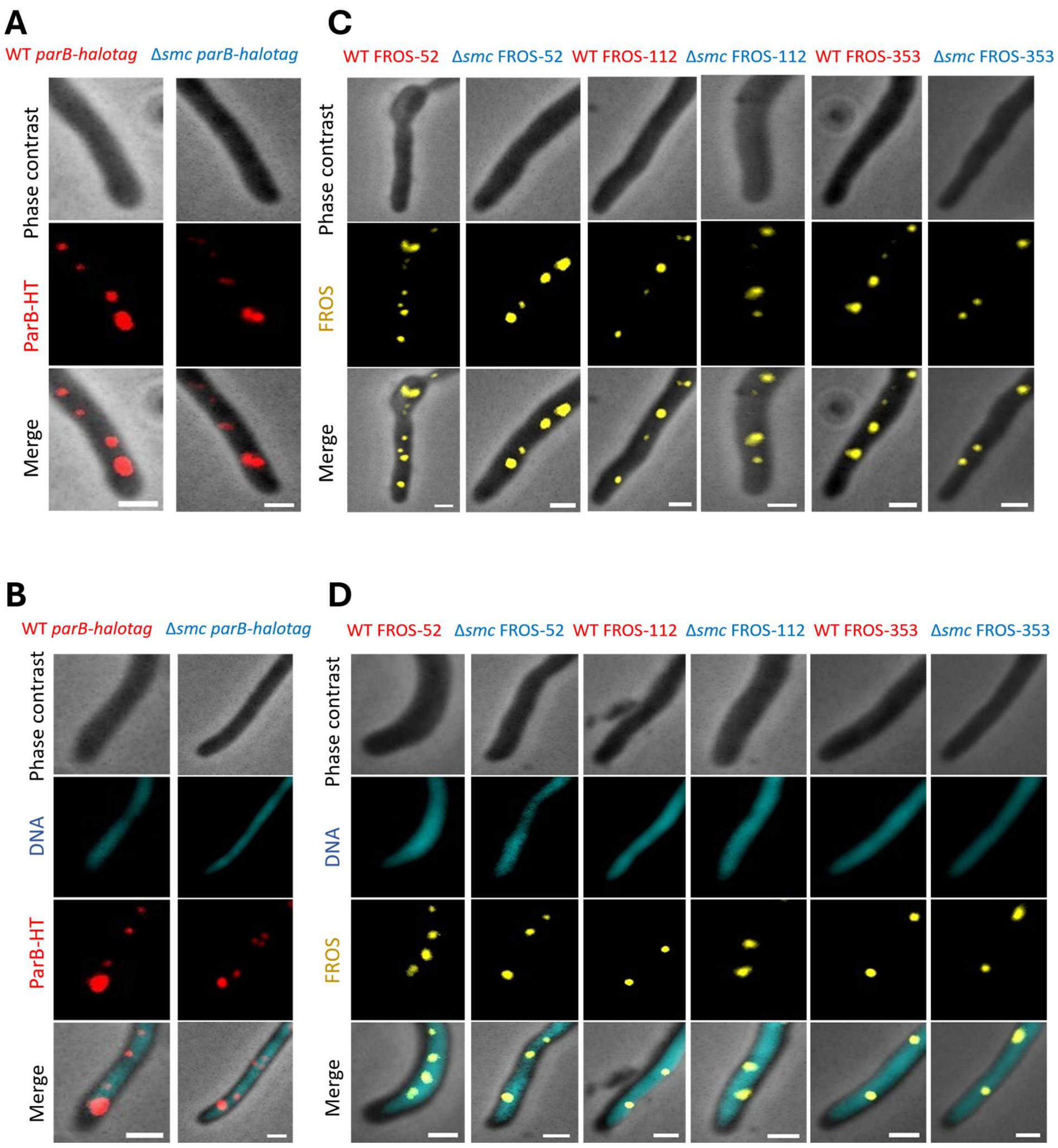

Fig. S8. Microscopy analyses of ParB-HT focus and FROS-52 focus appearance in the germ tube of the control and Δ*smc* strain. A. Representative microscopic images showing the apical ParB-HT complex in the wild type (KP006) and Δ*smc* (KP007) background. The top panel images show the phase-contrast image (grey, top panel), fluorescence of ParB-HT stained with TMR *direct ligand* (red, middle panel), and both channels overlaid. The bottom panel shows images of the apical ParB-HT complex in relation to the nucleoid in the wild-type (KP006) and Δ*smc* (KP007) backgrounds. The images show the phase-contrast image (grey, top panel), Hoechst33342-stained DNA (blue, top-middle panel), fluorescence of ParB-HT stained with TMR *direct ligand* (red, bottom-middle panel), and three channels overlaid (bottom panel). **C.** Representative microscopic images showing the apical FROS complex in the wild type: KP012 (WT, FROS-52), KP013 (WT, FROS-112), KP014 (WT, FROS-353) and Δ*smc*: KP015 (Δ*smc*, FROS-52), KP016 (Δ*smc*, FROS-112), KP017 (Δ*smc*, FROS-353) background. The images show the phase-contrast image (grey, top panel), the fluorescence of TetR-mVenus (FROS – yellow, middle panel) and

both channels overlaid (bottom panel). **D** Representative microscopic images showing the apical FROS complex in the wild type: KP012 (WT, FROS-52), KP013 (WT, FROS-112), KP014 (WT, FROS-353) and Δ*smc*: KP015 (Δ*smc*, FROS-52), KP016 (Δ*smc*, FROS-112), KP017 (Δ*smc*, FROS-353) background. The images show the phase-contrast image (grey, top panel), Hoechst33342-stained DNA (blue, top-middle panel), the fluorescence of TetR-mVenus (FROS – yellow, middle panel) and both channels overlaid (bottom panel). Hyphae from 5-hour cultures were analysed. Scale bar - 1 µm.
