# Supplementary material for: SMC ensures efficient chromosome replication and oriC positioning during Streptomyces spore germination
Source: Sci Rep. 2026 Mar 15;16:13557. doi: 10.1038/s41598-026-43107-5 (PMC13121528; doi:10.1038/s41598-026-43107-5)
Supplement: Supplementary file 2 — Supplementary Material 2 [file 41598_2026_43107_MOESM2_ESM.pdf]

**SMC Ensures Efficient Chromosome Replication and *oriC* Positioning During *Streptomyces* Spore Germination**

Katarzyna Pawlikiewicz[1], Agnieszka Strzałka[1], Agnieszka Nurek[1#], Magdalena Donczew[1&], Anna Gierlikowska[1&], Martyna Gongerowska-Jac[1], Marcin J. Szafran[1], Dagmara Jakimowicz[1*]

## SUPPLEMENARY INFORMATION

**Table S1. Plasmids and cosmids used in work**

| Plasmid/Cosmid | Characteristics | Source |
|---|---|---|
| **pSS170** | Integrative plasmid derivative pMS82(1), with a modified MCS site, integration site - attBΦBT1, $Hyg^R$, *oripBR322*, *oriT* (RP4) | Courtesy Dr. S. Schlimpert, John Innes Centre, Norwich, United Kingdom Britain (2) |
| **pSS170-$p_{tcp}$*halotag* (pK01)** | pSS170 carrying *halotag* gene under the control of $p_{tcp}$ promoter, integration site - attBΦBT1, $Hyg^R$, *ori* pBR322, *oriT* (RP4) | (3) |
| **pBAD *mvenus*** | Non-integrative plasmid, containing *mvenus* gene, 6xHis, T7-tag, $Amp^R$, *ori* pBR322, | (4) |
| **pLAU44** | Non-integrative plasmid, containing tandem tetO cassettes, $Gm^R$, $Amp^R$, *ori* pBR322, | (5) |
| **pOJ260** | Non-integrative, non-replicable plasmid in *Streptomyces* $Kan^R$, $Apr^R$, *ori* pBR322, *oriT* (RP4) | (6) |
| **H18*dnaN-egfp-apr*** | Modified cosmid containing a fragment of the *S. coelicolor* chromosome containing the *dnaN-EGFP* gene and Apra cassette, $Amp^R$, $Kan^R$ $Apr^R$ | (7) |
| **Sv-4-A09** | A cosmid containing a fragment of the *S. venezuelae* chromosome (3,947,666-3,987,790 of NZ_CP018074.1 genome) encompassing *dnaN* gene in the SuperCos-1 vector, $Amp^R$, $Kan^R$ | John Innes Centre, Norwich, United Kingdom |
| **Sv-5-B09** | A cosmid containing a fragment of the *S. venezuelae* chromosome (4,019,933-4,062,201 of NZ_CP018074.1 genome) encompassing *vnz_18215* gene in the SuperCos-1 vector, $Amp^R$, $Kan^R$ | John Innes Centre, Norwich, United Kingdom |
| **Sv-3-G09** | A cosmid containing a fragment of the *S. venezuelae* chromosome (4,048,114-4,090,725 of NZ_CP018074.1 genome) encompassing the | John Innes Centre, Norwich, United Kingdom |

| | | |
|---|---|---|
| | *vnz_18495* gene in the SuperCos-1 vector, $Amp^R$, $Kan^R$ | |
| **Sv-4-E11** | A cosmid containing a fragment of the *S. venezuelae* chromosome (4,295,185-4,336,803 of NZ_CP018074.1 genome) encompassing the *vnz_19695* gene in the SuperCos-1 vector, $Amp^R$, $Kan^R$ | John Innes Centre, Norwich, United Kingdom |
| **pSS170 $p_{nat}$*parAB-halotag* (pKP05)** | pSS170 carrying the *S. venezuelae parAB* operon (3,977,900-3,980,433 of NZ_CP018074.1 genome) wih *parB* in fusion with the *halotag* gene under the control of the native *parAB* promoter*,* integration site - attBΦBT1, $Hyg^R$, *ori* pBR322, *oriT* (RP4) | (3) |
| **pOJ260-52** | pOJ260 carrying a 1738 bp fragment (4,022,377 - 4,024,115 of NZ_CP018074.1 genome) encompassing the *S. venezuelae vnz_18215* gene, $Kan^R$, $Apr^R$, *ori* pBR322, *oriT* (RP4) | This work |
| **pOJ260-112** | pOJ260 carrying a 1855 bp fragment (4,085,139-4,086,994 of NZ_CP018074.1 genome) encompassing the *S. venezuelae vnz_18495* gene, $Kan^R$, $Apr^R$, *ori* pBR322, *oriT* (RP4) | This work |
| **pOJ260-353** | pOJ260 carrying a 1752 bp fragment (4,326,035-4,327,787 of NZ_CP018074.1 genome) encompassing *S. venezuelae vnz_19695* gene, $Kan^R$, $Apr^R$, *ori* pBR322, *oriT* (RP4) | This work |
| **pOJ260 52 *tetR-mvenus* (pKP21)** | pOJ260-52 carrying the *tetR-mvenus* gene under the control of the pS14 promoter and a 1721 bp fragment encompassing *S. venezuelae vnz_18215* gene, $Kan^R$, $Apr^R$, *ori* pBR322, *oriT* (RP4) | This work |
| **pOJ260 FROS-52 (pKP12)** | pOJ260-52 carrying the *tetR-mvenus* gene under the control of the pS14 promoter, 49 tandem *tetO* operator cassettes and a 1738 bp fragment encompassing the *S. venezuelae vnz_18215* gene, $Kan^R$, $Apr^R$, *ori* pBR322, *oriT* (RP4) | This work |
| **pOJ260 FROS-112 (pKP13)** | pOJ260-112 carrying the *tetR-mvenus gene* under the control of the pS14 promoter, 49 tandem *tetO* operator cassettes, and a 1855 bp fragment encompassing the *S. venezuelae vnz_18495* gene, $Kan^R$, $Apr^R$, *ori* pBR322, *oriT* (RP4) | This work |
| **pOJ260 FROS-353 (pKP14)** | pOJ260-353 carrying the *tetR-mvenus* gene under the control of the pS14 promoter, 49 tandem *tetO* operator cassettes and a 1752 bp fragment encompassing the *S. venezuelae vnz_19695* gene, $Kan^R$, $Apr^R$, ori pBR322, oriT (RP4) | This work |

| | | |
|---|---|---|
| **pFLUX** | pBluescript KS(+)derivative (*ori*pUC), *oriT* (RK2), FRT sites, carrying *luxCDABE* genes, $Apr^R$ | (8) |
| **pFLUXH** | pFLUX plasmid with hygromycin resistance cassette, $Hyg^R$ | (9) |
| **pFLUXH*crp*** | pFLUXH carrying *crp* gene promoter (3,662,194-3,66,703 of NZ_CP018074.1 genome) | This work |
| **pFLUXH*prs*** | pFLUXH carrying *prs* gene promoter (5,286850-5,287,355 of NZ_CP018074.1 genome) | This work |
| **pFLUXH*sigB*** | pFLUXH carrying *sigB* gene promoter (392,742-393,237 of NZ_CP018074.1 genome) | This work |
| **pFLUXH*bldD*** | pFLUXH carrying *bldD* gene promoter (1,237,806-1,237,294 of NZ_CP018074.1 genome) | This work |
| **pFLUXH*bldG*** | pFLUXH carrying *bldG* gene promoter (3,638,369-3,638,860 of NZ_CP018074.1 genome) | This work |

**Table S2. Bacterial strains used in this study**

| Strain | Characteristics | Source |
|---|---|---|
| | ***E. coli*** | |
| **DH5α** | F-, Φ80dlacZM15, recA1, *endA1, gyrA96, thi-1,hsdR17, (rk-, mk+), supE44, relA1, deoR, (lacZY*AargF) *U169* | Lab stock |
| **ET12567 /pUZ8002** | *dam13::TN9, dcm6, hsdM, hsdR, recF134, zjj201::TN10, galK2, galT22, ara14, lacY1, xyl5, leuB6, thi1, tonA31, rpsL136, hisG4, tsx78, mtli, glnV44*, F-, ($Cml^R$) Plasmid pUZ8002: *tra*, Kan $^R$,RP4 23 | (6) |
| | | |
| | ***S. venezuelae*** | |
| **WT** | Wild-type strain *Streptomyces venezuelae* in the *NRRL* culture collection database, deposited under NRRL number B-65442, genome NZ_CP018074.1 | (10) |
| **TM010 (Δ*smc*)** | Δ*smc* ::scar | (2) |
| **MD070 (*dnaN-egfp*)** | *dnaN-egfp* | This work |
| **KPAG01 (Δ*smc*, *dnaN-egfp)*** | TM010, *dnaN-egfp* | This work |
| **KP006 (Δ*parAB,* $p_{nat}$*parAB-halotag*)** | Δ*parAB* attBφBT1:: pSS170 $p_{nat}$*parAB-halotag* ($Hyg^R$) | (11) |
| **KP007 (Δ*smc*, Δ*parAB*, $p_{nat}$*parAB*-*halotag*)** | Δ*smc*Δ*parAB* attBφBT1:: pSS170$p_{nat}$*parAB-halotag* ($Apr^R$, $Hyg^R$) | (11) |
| **KP012 (WT, FROS-52)** | WT pOJ260-FROS-52 ($Apr^R$) | This work |
| **KP013 (WT, FROS-112)** | WT pOJ260-FROS-112 ($Apr^R$) | This work |
| **KP014 (WT, FROS-353)** | WT pOJ260-FROS-353 ($Apr^R$) | This work |
| **KP015 (Δ*smc*, FROS-52)** | TM010 pOJ260-FROS-52 ($Apr^R$) | This work |
| **KP016 (Δ*smc*, FROS-112)** | TM010 pOJ260-FROS-112 ($Apr^R$) | This work |
| **KP017 (Δ*smc*, FROS-353)** | TM010 pOJ260-FROS-353 ($Apr^R$) | This work |
| **KP021 (WT, *tetR-mvenus*)** | WT pOJ260-52-*tetR-mvenus* ($Apr^R$) | This work |

| | | |
|---|---|---|
| **WT pFLUXH** | WT attBφBT1:: pFLUXH (HygR) | This work |
| **WT pFLUXH*crp*** | WT attBφBT1:: pFLUXH*crp*(HygR) | This work |
| **WT pFLUXH*prs*** | WT attBφBT1:: pFLUXH*prs* (HygR) | This work |
| **WT pFLUXH*sigB*** | WT attBφBT1:: pFLUXH*sigB* (HygR) | This work |
| **WT pFLUXH*bldD*** | WT attBφBT1:: pFLUXH*bldD*(HygR) | This work |
| **WT pFLUXH*bldG*** | WT attBφBT1:: pFLUXH*bldG*(HygR) | This work |
| **Δ*smc* pFLUXH*crp*** | WT attBφBT1:: pFLUXH*crp*(HygR) | This work |
| **Δ*smc* pFLUXH*prs*** | WT attBφBT1:: pFLUXH*prs* (HygR) | This work |
| **Δ*smc* pFLUXH*sigB*** | WT attBφBT1:: pFLUXH*sigB* (HygR) | This work |
| **Δ*smc* pFLUXH*bldD*** | WT attBφBT1:: pFLUXH*bldD* (HygR) | This work |
| **Δ*smc* pFLUXH*bldG*** | WT attBφBT1:: pFLUXH*bldG* (HygR) | This work |

**Table S3. Oligonucleotides used in the work**

| Oligo-nucleotide | Sequence 5' → 3' | Application |
|---|---|---|
| **KP_71Fw** | TTAAGCCTAGGTACATCTCACGCGTCGGCCTTGACCTTGATGAGGCGGC | Cloning the $p_{S14}$ promoter and the *tetR* gene to pOJ260 52, pOJ26 112, pOJ260 353 and pOJ260 52 *tetR-mvenus* |
| **KP_71Rv** | CAGCTCCTCGCCCCCTTGCTCACGGATCCATCGTTATTCCTAGGCATCGAGGCCGCTTTCGCACTTTAGCT | |
| **KP_72Fw** | AGCTAAAGTGCGAAAGCGGCCTCGAGATGCCTAGGAATAACGATGGATGGATCCGTGAGCAAGGGCGAGGAGCTG | Cloning the fragment encoding the linker sequence and the *mvenus* gene to the pOJ260 52, pOJ26 112, pOJ260 353 and pOJ260 52 *tetR-mvenus* |
| **KP_72Rv** | ACGCTCACTGGTACCTTAATTAACACGCGTTTACTTGTACAGCTCGTCCATGC | |
| **KP_73Fw** | TGTACAAGTAAACGCGTTAATTAAGGTACCGAGGCTCTTAGCATGGAGTCTGAT | Cloning of the fragment containing the *tetO*49 operator cassette to pOJ260 52, pOJ26 112, pOJ260 353 and pOJ260 52 *tetR-mvenus* |
| **KP_81Rv** | ATCCTCTAGAGTCACCTGCAGCCCACCATGCCTGCTAGAGTGGCTT | |
| **KP_74Fw** | GATCCGCGGCCGCCCGCGCGCGATATCGGGGCACAAGGGGTACGTCCA | Cloning of the fragment (4,022,377 -4,024,115 of NZ_CP018074.1 genome) encompassing the *vnz_18215* gene into the pOJ260 vector |
| **KP_74Rv** | AAACAGCTATGACATGATTACGATGATGGCCCCCTTCGGGGGGGGGTCAGTCC | |
| **KP_75Fw** | ATCCGCGGCCGCGCGCGCGATATCGGCCTTAGTTGGTCCGGATTGC | Cloning of the fragment (4,085,139-4,086,994 of NZ_CP018074.1 genome) encompassing the *vnz_18495* gene into the pOJ260 vector |
| **KP_75Rv** | GAAACAGCTATGACATGATTACGAGCCCACCGGGGGGCTTCCCCCCCCCTT | |
| **KP_76Fw** | TCCGCGGCCGCCGCGGCGATATCGATCGACAAGGTCTACAACGGGGGC | Cloning of the fragment (4,326,035-4,327,787 of NZ_CP018074.1 genome) encompassing the *vnz_19695* gene into the pOJ260 vector |
| **KP_76Rv** | AAACAGCTATGACATGATTACGGGCTCCACCTGGGTTTCGTCCA | |
| **pSSseq_Fw** | AGGATCTTCACCTAGATCCTTTTGGT | Amplification of the pSS170 plasmid insert |
| **pSSseq_Rv** | GCCAGTGGTATTTATGTCAACACCGC | |
| **pOJ260seq_Fw** | GGTTTTCCCAGTCACGACGTTG | Amplification of the pOJ260 plasmid insert |
| **pOJ260seq_Rv** | GTGGAATTGTGAGCGGATAACAA | |
| **SvdnaN-gfpF** | GGCGTACAAGTACCTGATCATGCCGGTGCGCCTCAGCGGCCTGCCGGGCCCGGAGCTG | Construction of *dnaN-EGFP* fusion |

| | | |
|---|---|---|
| **SvdnaN-gfpR** | TACCCGGGTCCGAGCCTACGCCCGGACCCGGGTGCACACCCATATGTGTAGGCTGGAGCTGCTTC | |
| **gyr2_Fw** | ACAGGAAGGTCAGCAGCAG | Amplification of a fragment of the *S. venezuelae* chromosome 3,958,240 – 3,958,322 (near the *oriC* region), used for quantitative analysis of replication markers |
| **gyr2_Rv** | GCTCCGCTATCACAAGATCA | |
| **arg3_Fw** | CACCTGCGGATCTACAAGC | Amplification of a fragment of the *S. venezuelae* chromosome 1,320,274 – 1,320,351 (near the end of the right arm of the chromosome), used for quantitative analysis of replication markers |
| **arg3_Rv** | CCACTCCGACATCTCCTTG | |

| | | |
|---|---|---|
| **crp_pFLUXH_fw** | CTGAGTGACCAAAGGAGGCGGACAGGTCTGGTTGACCCGCAG | Amplification of the *crp* gene promoter (3,662,194-3,66,703of NZ_CP018074.1 genome) for insertion into the pFLUXH vector |
| **crp_pFLUXH_rv** | ATGATGAACGAGATCTTCTTCGTCATATGTTCTCTCCATACTGTCGACCTGC | |
| **prs_pFLUXH_fw** | CTGAGTGACCAAAGGAGGCGGACAGTCCAGCTCCGGATCTGTCTGAGCTGCGGGCACGT | Amplification of the *prs* gene promoter for introduction into the pFLUXH vector(5,286850-5,287,355 of NZ_CP018074.1 genome) |
| **prs_pFLUXH_rv** | ATGATGAACGAGATCTTCTTCGTCGTCATATGCACCTCCAAGGTGGCACA | |
| **sigB_pFLUXH_fw** | CTGAGTGACCAAAGGAGGCGGACAGTGCCGTTGCTCGGGAACAC | Amplification of the *sigB* gene promoter for introduction into the pFLUXH vector(392,742-393,237 of NZ_CP018074.1 genome) |
| **sigB_pFLUXH** | ATGATGAACGAGATCTTCTTCGTCATATGGTTCTCCTTTCACGTGTGTCCCGAACC | |
| **bldD_pFLUXH_fw** | CTGAGTGACCAAAGGGCGGACACTTCATCCGCGGTCGTCCCGGTACATCGT | Amplification of the *bldD* gene promoter for introduction into the pFLUXH vector(1,237,806-1,237,294 of NZ_CP018074.1 genome) |
| **bldD_pFLUXH_rv** | ATGATGAACGAGATCTTCTTCGTCATATGGCTCCCGGACGCTGTG | |
| **bldG_pFLUXH_fw** | CTGAGTGACCAAAGGAGGCGGACAGTCCAGGAGGGTGGTCAGG | Amplification of the *bldg* gene promoter for introduction into the pFLUXH vector(3,638,369-3,638,860 of NZ_CP018074.1 genome) |
| **bldG_pFLUXH_rv** | ATGATGAACGAGATCTTCTTCGTCATATGTCCTCCAGCACCTTGCTATCGAAC | |

## SUPPLEMENTARY METHODS

### Construction of mutant *S. venezuelae* strains

### Construction of *S. venezuelae dnaN-EGFP* and Δ*smc dnaN-EGFP* strains

To construct *dnaN-EGFP S. venezuelae* strains the Redirect procedure was applied (12). The *egfp-apr* cassette was PCR amplified using SvdnaN-gfpF and SvdnaN-gfpR primers and H18*dnaN-egfp-apr* cosmid (7) as the template. Using homologous recombination, an *egfp-apr* cassette was inserted into cosmid Sv-4-A09, resulting in the *dnaN-egfp-apr* cosmid. The modified cosmid was verified by restriction digestion and then introduced into the ET12567/pUZ8002 *E. coli* strain, which was used for conjugation with spores of the *S. venezuelae* wild type and Δ*smc* strain (TM010). Conjugants sensitive to kanamycin and resistant to apramycin were selected. The obtained strains were verified by PCR using chromosomal DNA as the template and by Western Blotting with anti-EGFP antibodies.

### Construction of *S. venezuelae* strains with *oriC*-labelling: KP012 (WT, FROS-52), KP013 (WT, FROS-112), KP014 (WT, FROS-353), KP015 (Δ*smc*, FROS-52), KP016 (Δ*smc*, FROS-112), KP017 (Δ*smc*, FROS-353) and KP021 (WT, *tetR-mvenus*).

Strains with *oriC*-labelled producing TetR-mVenus and containing repeated *tetO* sequences near the *oriC* were prepared by single crossing-over recombination of pOJ260 derivatives ($Apr^R$, $Kn^R$). pOJ260 derivative contained the *tetR-mvenus* gene under the control of a strong constitutive promoter S14 (pS14) and a cassette encompassing 49 *tetO* repeats as well as fragments homologous to selected regions of the *S. venezuelae* chromosome located 52 kb (pOJ-FROS-52), 112 kb (pOJ-FROS-112), and 353 kb (pOJ-FROS-353) from the *oriC* on the *Streptomyces* chromosome, enabling single crossing over.

To construct the pOJ260-52 vector, a fragment corresponding to the region located 52 kb from *oriC*, encompassing the *vnz_18215* gene, was amplified using Sv_5-B09 cosmid as the template and KP_74Rv and KP_74Fw primers, yielding a 1738 bp product. To construct the pOJ260-112 vector, a fragment corresponding to the region located 112 kb from *oriC*, encompassing the *vnz_18495* gene, was amplified using the Sv_3-G09 cosmid as the template and KP_75Fw/KP_75Rv primers, yielding a 1855 bp product. To construct the pOJ260-353 vector, a fragment corresponding to the region located 353 kb from *oriC*, encompassing the *vnz_19695* gene, was amplified using Sv_4-E11 cosmid as the template and *KP_76Fw/KP_76Rv* primers, yielding a 1752 bp product. All PCR products were cloned by the SLIC method into a pOJ260 plasmid digested with EcoRI. Reaction mixtures were used to transform *E.coli* DH5α competent cells, and transformants resistant to apramycin and kanamycin were selected. The obtained constructs were verified by PCR reaction using primers pOJ260seq_Fw and pOJ260seq_Rv, and the obtained products were sequenced. The obtained vectors pOJ260-52, pOJ260-112, and pOJ260-353 were further modified by the insertion of *tetR-mVenus* and the *tetO* cassette.

To this end, the *tetR* gene with the S14 (pS14) promoter region was amplified using the pSS170-$p_{tcp}$*halotag* vector as the template with KP_71Fw and KP_71Rv primers, delivering a 762 bp product. In parallel, the *mVenus* gene was amplified using KP_72Fw and KP_72Rv primers and the pBAD-*mVenus* plasmid as the template, with the addition of a linker coding sequence (Met-Pro-Arg-Asn-Asn-Asp-Gly-Ser), yielding a 788 bp product. At the same time, a fragment comprising 49 *tetO* cassettes was amplified with KP_73Fw and KP_81Rv primers using pLAU44 plasmid as the template, resulting in a 2261 bp product. The obtained three fragments were cloned by Gibson Assembly into pOJ260-52, pOJ260-112, and pOJ260-353 vectors. To prepare the control plasmid, *tetR* and *mVenus* (but not the *tetO* cassette) were cloned by Gibson Assembly into the pOJ260-52 vector. Reaction mixtures were used to transform *E. coli* DH5α competent cells, and transformants resistant to apramycin and kanamycin were selected. The obtained constructs were verified by PCR reaction using pOJ260seq_Fw and pOJ260seq_Rv primers, and the obtained products were sequenced. The obtained plasmids (pOJ260-FROS-52, pOJ260-FROS-112, pOJ260-FROS-353, pOJ260-52 *tetR-mvenus*) were introduced into ET12567/pUZ8002 *E. coli* and conjugated to wild type and Δ*smc* (TM010) *S. venezuelae* strain. Conjugants resistant to apramycin and kanamycin were selected. The obtained strains were verified using PCR performed on the chromosomal DNA as the template, and Western blotting was used with anti-EGFP antibodies.

**Construction of the *S. venezuelae lux* reporter strains**

pFLUXH plasmid derivatives containing the *lux* operon were used for reporter gene studies. To construct pFLUXH plasmid derivatives pFLUXH was digested with the NdeI, dephosphorylated and purified. The promoter regions of the *crp, prs*, *sigB, bldG and bldD* genes were amplified using chromosomal DNA as the template and the following primer pairs: crp_pFLUXH_fw and crp_pFLUXH_rv, prs_pFLUXH_fw and prs_pFLUXH_rv, and sigB_pFLUXH_fw and sigB_pFLUXH_rv, bldB_pFLUXH_fw and bldB_pFLUXH_rv and bldG_pFLUXH_fw and bldG_pFLUXH_rv. PCR products encompassing the promoter regions of *crp* (509 bp), *prs* (505 bp), *sigB* (495 bp), *bldD* (488 bp) and *bldG* (490 bp) were cloned into the pFLUXH vector (modified pFLUX, $Hyg^R$) using the SLIC method. The reaction mixture was used to transform *E. coli* DH5α competent cells, and transformants resistant to hygromycin were selected. The clones were verified by PCR, digestion and sequencing. Obtained plasmids: $pFLUXH_{sigB}$, $pFLUXH_{prs}$, $pFLUXH_{crp}$, $pFLUXH_{bldD}$, $pFLUXH_{bldG}$ and the control vector pFLUXH were used to modify wild type and Δ*smc* (TM010) *S. venezuelae* strains by intergeneric conjugation. The conjugants were selected with nalidix acid and hygromycin.

**Lux reporter gene analyses**

Spore suspensions of selected *S. venezuelae* strains were diluted with Milli-Q water to an $OD_{600}$ of 0.3. Then, 200 µL of liquid MYM medium without antibiotics were applied to the wells of a 96-well flat-bottom white polystyrene plate (Corning) with a transparent bottom and inoculated with 20 µL of diluted spores. $OD6_{00}$ and culture bioluminescence were measured every 20 minutes during cultures at 3 × 3 points per well in the Infinite 200Pro plate reader (Tecan) at 30 °C with shaking (2 mm amplitude). Five repetitions were performed and the results were analysed in Excel.

**Measuring the growth rate *of S. venezuelae* using the Bioscreen C reader**

Growth rate analyses were carried out using the BioscreenC apparatus (OY Growth Curves Ab Ltd - ALAB). Briefly, *S. venezuelae* were cultured in flat-bottomed transparent Honeycomb plates. *S. venezuelae* spores were diluted in 10% glycerol to achieve an $OD_{600}$ of 0.05, and then the suspension was diluted 300-fold in MYM liquid medium. 300 µl of the spore suspension was pipetted into wells of a 100-well plate, and 300 µl of sterile MYM medium was used as a control. All experiments were performed in 3 independent biological and technical replicates. Cultures were carried out in the Bioscreen C automated microorganism growth analyser for 24 - 48 h, at 30°C with medium shaking (250 rpm). Culture growth was monitored by optical density ($OD_{600}$) measurement every 20 minutes throughout the experiment. Data was collected using BioScreener 3.0.0 software. Based on the results obtained, growth curves were plotted using the R programming language.

**Preparation of *S. venezuelae* cell lysates and SDS PAGE**

To prepare cell lysates, biomass from a liquid culture of *S. venezuelae* or *E. coli* was centrifuged, rinsed twice with cold PBS buffer (volume equal to the culture, centrifugation for 5 min at 5000 rpm and 4°C), and resuspended in PBS buffer (200 µL for every 5 mL of initial culture). Samples were sonicated using the Sonics Vibra Cell Ultrasonic Liquid at 50% amplitude for 2x40 s. The samples were then centrifuged (10 min, 10000 rpm, 4°C). The resulting supernatant was transferred to new tubes, and the protein concentration was determined by the Bradford method. Lysates were used for SDS-PAGE analysis and Western *blotting*, with samples prepared to contain the same amount of total protein (10-30 µg). Protein separation was performed using a standard procedure and followed by immunoblotting for the detection of individual proteins. For immunoblotting, protein transfer to the membrane was performed in the Pierce Power Blotter (ThermoFisher Scientific). The membrane was incubated in a 5% blocking solution (skimmed milk) overnight at 4°C and next for one h mouse monoclonal anti EGFP antibody (Invitrogen MA1-952) diluted 1000X in TBST buffer (Tris-HCl pH 7.5 10 mM, NaCl 150

mM, Tween 20 0.1), washed, and incubated for one h with anit mouse IgG antibody conjugated with horseradish peroxidase (Santa Cruz Biotechnology) diluted 5000 in TBST buffer. The membrane was then washed, and a chemiluminescence reaction was used to detect HRP. For this purpose, the membrane was placed in a solution containing luminol for the detection of horseradish peroxidase and then read on the Bio-Rad ChemiDocTM XRS apparatus, using the Imaging System.

**Bibliography**


1. Gregory MA, Till R, Smith MCM. Integration Site for Streptomyces Phage □ BT1 and Development of Site-Specific Integrating Vectors. J Bacteriol. 2003;185(17):5320–3.
2. Szafran MJ, Małecki T, Strzałka A, Pawlikiewicz K, Duława J, Zarek A, et al. Spatial rearrangement of the Streptomyces venezuelae linear chromosome during sporogenic development. Nat Commun. 2021;12(1):5222.
3. Pawlikiewicz K, Strzałka A, Majkowski M, Duława-Kobeluszczyk J, Szafran MJ, Jakimowicz D. SMC modulates ParB engagement in segregation complexes in streptomyces. Nature Communications . 2025 Dec 1;16(1).
4. Nagai T IKPEKMMKMA. A variant of yellow fluorescent protein with fast and efficient maturation for cell-biological applications.  2002;20(1):87-90. doi:10.1038/nbt0102-87. Nat Biotechnol. 2002;20(1):87–90.
5. Lau IF, Filipe SR, Søballe B, Økstad OA, Barre FX, Sherratt DJ. Spatial and temporal organization of replicating Escherichia coli chromosomes. Mol Microbiol. 2003 Aug;49(3):731–43.
6. Kieser T, Bibb MJ, Buttner MJ, Chater KF, Hopwood DA. Practical Streptomyces Genetics. John Innes Centre Ltd. 2000;529.
7. Ruban-Ośmiałowska B, Jakimowicz D, Smulczyk-Krawczyszyn A, Chater KFKF, Zakrzewska-Czerwińska J. Replisome localization in vegetative and aerial hyphae of Streptomyces coelicolor. J Bacteriol [Internet]. 2006;188(20):7311–6.
8. Craney A, Hohenauer T, Xu Y, Navani NK, Li Y, Nodwell J. A synthetic luxCDABE gene cluster optimized for expression in high-GC bacteria. Nucleic Acids Res [Internet]. 2007;35(6):e46–e46.
9. Szafran MJMJ, Gongerowska M, Gutkowski P, Zakrzewska-czerwin J, Zakrzewska-Czerwińska J, Jakimowicz D. The Coordinated Positive Regulation of Topoisomerase Genes Maintains Topological Homeostasis in Streptomyces coelicolor. J Bacteriol. 2016;198(21):3016–28.
10. Gomez-Escribano JP, Holmes NA, Schlimpert S, Bibb MJ, Chandra G, Wilkinson B, et al. Streptomyces venezuelae NRRL B-65442: Genome sequence of a model strain used to study morphological differentiation in filamentous actinobacteria. J Ind Microbiol Biotechnol. 2021 Dec 1;48(9).
11. Pawlikiewicz K, Strzałka A, Majkowski M, Duława-Kobeluszczyk J, Szafran M, Jakimowicz D. SMC modulates ParB engagement in segregation complexes in <em>Streptomyces</em> bioRxiv [Internet]. 2024 Jan 1;16(1):2024.10.17.618854. Available from: http://biorxiv.org/content/early/2024/10/17/2024.10.17.618854.abstract
12. Gust B, Chandra G, Jakimowicz D, Yuqing TIAN, Bruton CJCJ, Chater KFKFC. Red-Mediated Genetic Manipulation of Antibiotic-Producing Streptomyces. Adances in Applied Microbiology. 2004;54:107–28.
